# Prevalence of undiagnosed HIV among children in South Africa, Côte d'Ivoire and Zimbabwe: a model‐based analysis to inform paediatric HIV screening programmes

**DOI:** 10.1002/jia2.26045

**Published:** 2022-12-15

**Authors:** Nicole C. McCann, Tijana Stanic, Martina Penazzato, Clare F. Flanagan, Elaine J. Abrams, Caitlin M. Dugdale, Leigh F. Johnson, Anne M. Neilan, Mary‐Ann Davies, Kenneth A. Freedberg, Patricia Fassinou, Meg Doherty, Shaffiq Essajee, Angela Mushavi, Djøra I. Soeteman, Andrea L. Ciaranello

**Affiliations:** ^1^ Medical Practice Evaluation Center Department of Medicine Massachusetts General Hospital Boston Massachusetts USA; ^2^ Global HIV Hepatitis and STI Programme World Health Organization Geneva Switzerland; ^3^ ICAP at Columbia University Mailman School of Public Health Columbia University New York USA; ^4^ Department of Pediatrics Vagellos College of Physicians and Surgeons Columbia University New York USA; ^5^ Division of Infectious Diseases Massachusetts General Hospital Boston Massachusetts USA; ^6^ Centre for Infectious Disease Epidemiology and Research School of Public Health and Family Medicine Faculty of Health Sciences University of Cape Town Cape Town South Africa; ^7^ Division of Pediatric Outcomes Research Massachusetts General Hospital Boston Massachusetts USA; ^8^ Division of General Internal Medicine Department of Medicine Massachusetts General Hospital Boston Massachusetts USA; ^9^ Harvard University Center for AIDS Research Boston Massachusetts USA; ^10^ Department of Health Policy and Management Harvard School of Public Health Boston Massachusetts USA; ^11^ Elizabeth Glaser Pediatric AIDS Foundation Abidjan Côte d'Ivoire; ^12^ UNICEF New York USA; ^13^ Ministry of Health and Child Care Harare Zimbabwe; ^14^ Center for Health Decision Science Harvard T.H. Chan School of Public Health Boston Massachusetts USA

**Keywords:** Africa < region, HIV care continuum, modelling, paediatrics, testing, vertical transmission

## Abstract

**Introduction:**

To improve the diagnosis and survival of children living with HIV (CLWH), the World Health Organization recommends testing approaches beyond traditional infant HIV testing programmes. Information about undiagnosed HIV prevalence among children of varying ages in the general population is needed to guide innovative national/subnational case‐finding and testing approaches.

**Methods:**

We used the Cost‐Effectiveness of Preventing AIDS Complications (CEPAC)‐Pediatric model to estimate the prevalence of undiagnosed HIV in 2‐, 5‐ and 10‐year‐old children in South Africa, Côte d'Ivoire and Zimbabwe in 2018. We simulated cohorts of children born in 2008 (10‐year‐olds), 2013 (5‐year‐olds) and 2016 (2‐year‐olds). Country‐/year‐specific inputs for pregnant/breastfeeding women included: HIV prevalence (4.2–32.3%), HIV incidence (0.03–0.24%/month), knowledge of HIV status (27–89%) and antiretroviral drug coverage (36–95%). Paediatric inputs included early infant testing coverage (6–95%) and breastfeeding duration (0–20 months). We projected the proportion of surviving CLWH in whom HIV remained undiagnosed and the undiagnosed HIV prevalence among surviving children of each age in the general population. For children born in 2016, we projected survival and diagnosis of all CLWH through 2026. We conducted sensitivity analyses on model parameters.

**Results:**

In 2018, the projected proportion of surviving CLWH whose HIV remained undiagnosed in South Africa/Côte d'Ivoire/Zimbabwe was 44.2%/55.8%/52.9% among 2‐year‐old CLWH; 29.0%/37.8%/33.2% among 5‐year‐old CLWH; and 18.3%/25.4%/23.1% among 10‐year‐old CLWH. Projected general population undiagnosed HIV prevalence in South Africa/Côte d'Ivoire/Zimbabwe was 0.44%/0.32%/0.68% among 2‐year‐olds; 0.25%/0.17%/0.41% among 5‐year‐olds; and 0.24%/0.14%/0.38% among 10‐year‐olds. Among all CLWH born in 2016, 50–54% were projected to die without HIV diagnosis (and subsequently without treatment) within 10 years after birth; 80–85% of these deaths occurred in the first 2 years.

**Conclusions:**

Projected population‐level undiagnosed HIV prevalence is low and sharply decreases after age 2, with more CLWH dying than being diagnosed. Despite low undiagnosed prevalence in the general population of older children, we project that a large proportion of CLWH remain undiagnosed, suggesting that innovative strategies targeting untested children of all ages outside of health facility settings should be prioritized. Programmes could consider routine testing of the general population of children below 2 in all settings and children of all ages in high‐prevalence settings.

## 1 INTRODUCTION

Great progress has been made in reducing HIV infections among children in the past decade. Following the scale‐up of HIV testing and universal antiretroviral therapy (ART) for pregnant/breastfeeding women, from 2010 to 2021, estimated numbers of new children living with HIV (CLWH) globally dropped from 320,000 (95% CI: 220,000–480,000) to 160,000 (95% CI: 110,000–230,000) [1]. Despite this progress, only 52% (95% CI: 42–65%) of CLWH received ART and 98,000 (95% CI: 67,000–140,000) died from HIV/AIDS in 2021 [2, 3]. Poor paediatric outcomes may be in part due to missed opportunities for HIV testing in children, with delays in diagnosis and treatment leading to high mortality early in life [4].

The World Health Organization (WHO) recommends that children with known HIV exposure be tested for HIV at 6 weeks of age [5]. However, infant testing programmes may miss children born to women with HIV who are not engaged in care and/or do not know their status; these children face higher HIV transmission and disease progression risks while undiagnosed [4, 6]. Additionally, although follow‐up testing is recommended at older ages (6–18 months), uptake is often low among children who test negative at 6 weeks (e.g. 24–45% in South Africa), increasing the risk that children who acquire HIV through breastfeeding are undiagnosed [5, 7–12]. To reach children missed by traditional infant testing, the WHO further recommends provider‐initiated testing and counselling (PITC) in paediatric care settings in addition to testing programmes for infants known to be born to women with HIV. In low‐burden settings, testing is recommended for children with symptoms suggestive of HIV, including TB. In high‐burden settings, the WHO recommends testing in inpatient and malnutrition services (strong recommendation), and outpatient and immunization clinics (conditional recommendation). In all settings, testing children of adults with HIV (index testing) is suggested (good practice statement) [5, 13].

Although paediatric PITC is recommended, it is less commonly implemented than 6‐week infant testing across sub‐Saharan Africa, possibly due to anticipated low yield due to competing priorities in busy health facilities, limited human capacity, cost concerns in settings where paediatric HIV prevalence is considered low and lack of testing algorithms for specific settings [5, 13]. Undiagnosed HIV prevalence among children in the general population without indications to seek medical care (e.g. household‐ or school‐based populations) is unknown, as children with the highest HIV acquisition risk often are least likely to present to programmes with testing and data collection. We aimed to estimate undiagnosed HIV prevalence among children at various ages in South Africa, Côte d'Ivoire and Zimbabwe using a simulation model, to inform the design and potential impact of various PITC interventions.

## 2 METHODS

### 2.1 Overview

We used the Cost‐Effectiveness of Preventing AIDS Complications‐Pediatric (CEPAC‐P) model, a validated Monte Carlo microsimulation model of HIV disease [14], to project undiagnosed HIV prevalence in 2‐, 5‐ and 10‐year‐old children in South Africa, Côte d'Ivoire and Zimbabwe in 2018. For each country, we modelled birth cohorts of infants born in 2008 (10‐year‐olds), 2013 (5‐year‐olds) and 2016 (2‐year‐olds). We modelled children surviving to these specific ages to reflect the impacts of changes in WHO HIV treatment guidelines that occurred in 2008, 2013 and 2016 and to add to limited epidemiological data on undiagnosed HIV prevalence among children over age 2 [15]. We selected three countries that spanned a range of HIV epidemic types: South Africa with high HIV prevalence and high antiretroviral drug (ARV) coverage in pregnant/breastfeeding women, Côte d'Ivoire with low prevalence and lower ARV coverage and Zimbabwe with intermediate prevalence and high ARV coverage. We applied HIV prevalence and incidence, knowledge of HIV status and ARV coverage in pregnant/breastfeeding women, as well as infant/paediatric testing coverage and breastfeeding duration for each country and calendar year. For each modelled cohort, we projected the probability that children surviving to 2018 would have undiagnosed HIV.

### 2.2 CEPAC‐P model

In CEPAC‐P, children are simulated in monthly cycles starting from birth. CEPAC‐P projects monthly HIV acquisition, HIV‐related and non‐HIV‐related mortality and life expectancy (Supplement, pp. 3–5). ARV availability and regimen (e.g. single‐dose nevirapine, short‐course zidovudine or three‐drug combination ART) used during pregnancy determine vertical transmission risk, modelled as a one‐time intrauterine/intrapartum risk and a monthly risk while breastfeeding (Table 1). All simulated children experience monthly age‐stratified risks of non‐HIV‐related mortality; CLWH face additional monthly risks of opportunistic infections (OI), death from OI and HIV‐related mortality, stratified by age and CD4%/CD4 count (Figure S1).

**Table 1:** Model inputs: HIV care continuum parameters

South Africa				
HIV care continuum parameter^b^	2008	2013	2016	Source
HIV prevalence in pregnant women, %	32.3	32.3	32.3	[17, 18]
HIV incidence during breastfeeding, monthly, %	0.24	0.24	0.24	[21]
Knowledge of status during pregnancy and breastfeeding, %	74	89	89	[23, 24, 50, 51, 52]
ARV coverage in pregnant/breastfeeding women, %	73	90	95	[27, 28, 29]
Infant/paediatric testing coverage at 6 weeks (birth + 10‐weeks in 2016)/6 months/18 months, %	36/0/0	76/0/0	95/25/22	[8, 9, 17, 30, 32]
Probability of diagnosis after presentation with severe OI,^a^ %	20^a^	20^a^	47	[36]
Proportion breastfeeding, % (mean duration, months)				
Unknown HIV status	82 (17)	66 (12)	66 (12)	[22, 23]
Known HIV status	0 (0)	66 (6)	66 (6)	

Côte d'Ivoire				
HIV care continuum parameter	2008	2013	2016	Source
HIV prevalence in pregnant women, %	5.9	4.7	4.2	[16]
HIV incidence during breastfeeding, monthly, %	0.04	0.03	0.03	[16, 19–22]
Knowledge of status during pregnancy and breastfeeding, %	27	32	57	[25, 51, 53–55]
ARV coverage in pregnant/breastfeeding women, %	41	75	73	[27, 28, 29]
Infant/paediatric testing coverage at 6 weeks/9 months/18 months, %	6/0/0	12/0/0	39/26/22	[9, 10, 30]
Probability of diagnosis after presentation with severe OI,^a^ %	20^a^	20^a^	47	[36]
Proportion breastfeeding, % (mean duration, months)				
Unknown HIV status	98 (20)	97 (19)	97 (19)	[33]
Known HIV status	98 (20)	97 (19)	97 (19)	

Zimbabwe				
HIV care continuum parameter	2008	2013	2016	Source
HIV prevalence in pregnant women, %	20.3	18.8	17.9	[16]
HIV incidence during breastfeeding, monthly, %	0.15	0.14	0.14	[16, 19–22]
Knowledge of status during pregnancy and breastfeeding, %	47	73	84	[26, 51, 52, 56, 57]
ARV coverage in pregnant/breastfeeding women, %	36	78	93	[27, 28, 29]
Infant/paediatric testing coverage at 6 weeks/9 months/18 months, %	6/0/0	57/0/0	71/26/22	[9, 10, 30, 31]
Probability of diagnosis after presentation with severe OI,^a^ %	20^a^	20^a^	47	[36]
Proportion breastfeeding, % (mean duration, months)				
Unknown HIV status	98 (17)	94 (17)	94 (17)	[26, 33]
Known HIV status	98 (17)	94 (17)	94 (17)	

Abbreviations: **ARV**, antiretroviral drug; **CLWH**, children living with HIV; **HIV,** human immunodeficiency virus; **OI**, opportunistic infection.

^a^ Data for probability of diagnosis after OI in 2016 are from Feucht et al. *AIDS Care* 2016 [36]; due to lack of data in earlier years, this parameter for 2008 and 2013 was calibrated to Thembisa model estimates of proportions of CLWH who remain undiagnosed [37] (see Supplement, pp. 3).

^b^ An expansion of the inputs shown in this table and their sensitivity analysis ranges, as well as additional inputs and sources, is shown in the Supplement (Table S1).

CLWH can be diagnosed through early infant testing (6‐week or birth and 10‐week) and later paediatric testing. Children can also be diagnosed through presentation to care with severe OI and subsequent testing. Upon confirmation of HIV, children experience a probability of linking to HIV care and initiating ART. Once on ART, they face a probability of initial virologic suppression, with a resulting CD4 rise, and subsequently a risk of monthly treatment failure. If HIV remains undiagnosed, individuals are subject to a “natural history” of untreated HIV disease (CD4 decline, OI and HIV‐related mortality). Additional details about CEPAC‐P are in the Supplement, and https://www.massgeneral.org/medicine/mpec/research/cpac‐model.

### 2.3 Model input parameters

Model inputs were derived during 2018–2022. To reflect children born in 2008, 2013 and 2016 in South Africa, Côte d'Ivoire and Zimbabwe, we simulated cohorts of children using year‐ and country‐specific data. We used demographic survey data, UNAIDS/UNICEF reports and published literature to reflect the HIV care continuum for pregnant/breastfeeding women and infants in each country and year, including: HIV prevalence in pregnant/breastfeeding women (4.2–32.3%) [16, 17, 18]; HIV incidence during breastfeeding (0.03–0.24%) [16, 17, 18, 19, 20, 21, 22]; knowledge of HIV status in pregnant/breastfeeding women (27–89%) [23, 24, 25, 26]; ARV coverage in pregnant/breastfeeding women with known HIV status (36–95%) [27, 28, 29]; early infant HIV testing coverage (6–95%) [17, 30–32]; and breastfeeding duration (0–20 months, depending on country/year and HIV status) [22, 26, 33] (Table 1 and Table S1). Based on WHO guidelines, early infant testing occurred at 6 weeks of age across all settings, except South Africa in 2016, which included birth and 10‐week infant testing per national guidelines [5, 9, 34]. Later paediatric testing, at 6 or 9 and 18 months, occurred in all settings in 2016 per WHO guidelines and country‐level data [5, 9, 10, 35]. Paediatric testing coverage upon presentation to care with OI was 20% in 2008 and 2013 and 47% in 2016 (Table 1 and Supplement, pp. 3) [36].

ARV regimens for pregnant/breastfeeding women were derived from published data and guidelines from relevant years. In the 2013 and 2016 birth cohorts, ART was used for all pregnant/breastfeeding women. In the 2008 birth cohort, ART was used in women with CD4 ≤200, and short‐course zidovudine and/or single‐dose nevirapine were used for women with CD4>200 (Table S1). We used ARV regimen‐specific peripartum and postpartum vertical transmission risks derived from a literature review [6]. Vertical transmission risks were highest for those not receiving any ARVs and lowest for those receiving ART. Women with chronic HIV were more likely to know their HIV status and initiate ARVs compared with those acutely infected. Among women on ART, risks were higher for those who initiated during pregnancy compared with those who initiated pre‐conception (Table 1 and Table S1).

CEPAC‐P is calibrated to published survival data of CLWH, with and without paediatric ART (Table S1) [14]. For this analysis, we further calibrated to country‐specific vertical transmission estimates (Tables S2 and S3, and Supplement, pp. 3).

### 2.4 Model outcomes

We projected early (intrauterine/intrapartum) vertical transmission, total (intrauterine/intrapartum/postpartum) vertical transmission by end of breastfeeding and survival among 2008, 2013 and 2016 birth cohorts in each country. For each calendar‐year birth cohort, we reported (1) the proportion of surviving CLWH with undiagnosed HIV, and (2) the proportion of children surviving to 2018 with undiagnosed HIV among the general population (“undiagnosed HIV prevalence”). We also projected the absolute number of undiagnosed CLWH of each age in South Africa [37]. To determine the relative contributions of treated and untreated pregnant/breastfeeding women with HIV to population‐level vertical transmission, we calculated the proportion of vertical transmission attributable to pregnant/breastfeeding women on and off ARVs. To estimate undiagnosed HIV prevalence beyond 2018, we projected survival among children born in 2016 acquiring HIV during their lifetime and projected the number of children anticipated to be in four mutually exclusive categories over 10 years: (1) those alive with undiagnosed HIV; (2) those alive with diagnosed HIV (diagnosed through infant/paediatric testing or after the presentation to care with OI); (3) those who had died with undiagnosed HIV; and (4) those who had died with diagnosed HIV.

### 2.5 Sensitivity analyses

In one‐way sensitivity analyses, we varied model input parameters to investigate how variations would impact undiagnosed HIV prevalence among 2‐year‐olds in 2018. We varied HIV care continuum inputs among pregnant/breastfeeding women and children described in *Model input parameters* and vertical transmission risks across plausible ranges (Table S1). Additionally, we varied paediatric natural history inputs, including HIV‐related mortality and probability of OIs, decreasing these parameters to 10% and 50% of their base‐case values (Table S1) [15]. In multi‐way sensitivity analyses, we simultaneously varied service delivery parameters that most impacted results in one‐way sensitivity analyses: knowledge of HIV status in pregnant/breastfeeding women, ARV coverage in pregnant/breastfeeding women and paediatric HIV testing coverage upon presentation to care with OI.

### 2.6 Ethical statement

The research was covered by a protocol approved by MGH IRB. Informed consent was not needed; we synthesized published data.

## 3 RESULTS

### 3.1 Projected total vertical transmission through 2018

**Figure 1:**
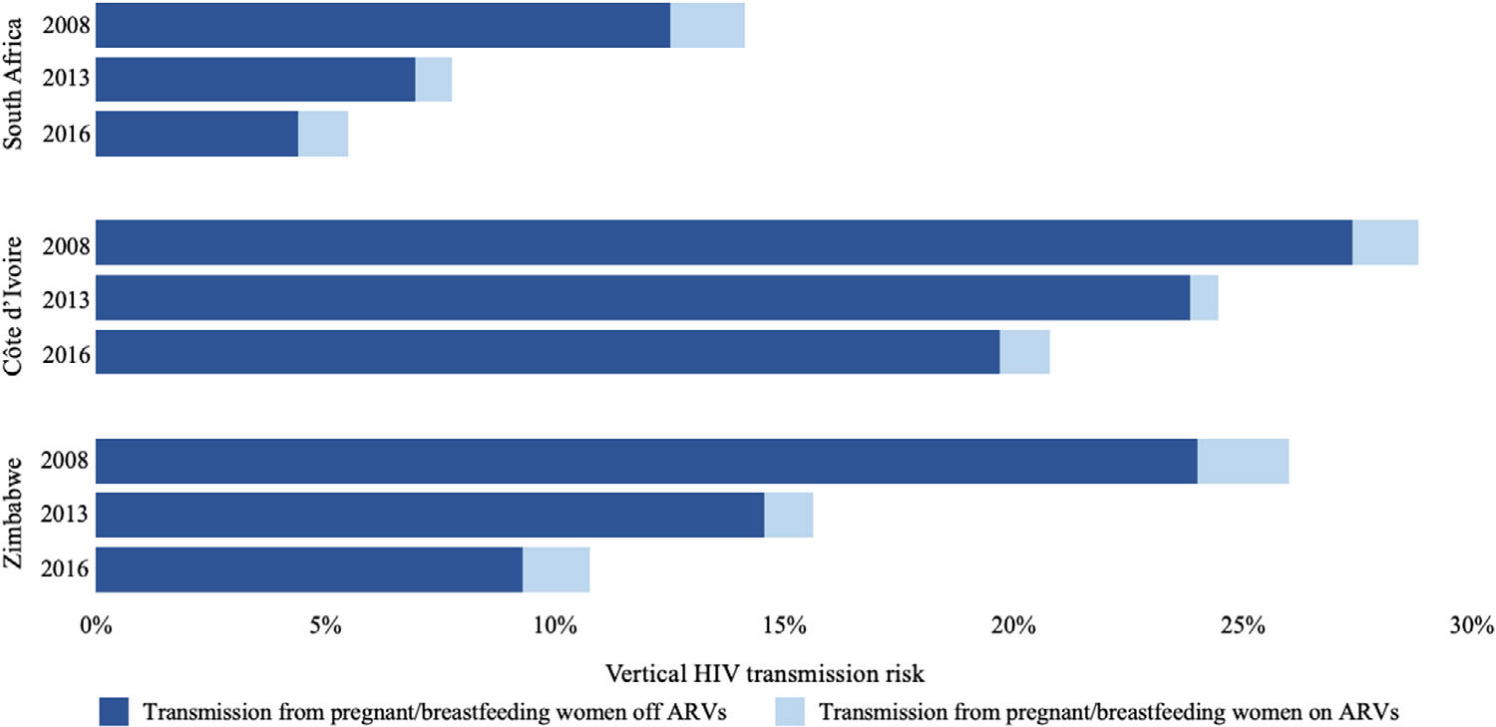
Projected vertical transmission by country, year and ARV status. Total projected vertical transmission by country, year and ARV status. On the y‐axis, South Africa, Côte d'Ivoire and Zimbabwe are shown, stratified by the 2008, 2013 and 2016 birth cohorts. Each bar represents the total vertical transmission (defined as the total number of children who acquire HIV/total exposed to HIV) per year. The dark blue represents the vertical transmission attributable to pregnant/breastfeeding women off ARVs, who either did not know their HIV status or did know their status, but did not initiate ARVs. The light blue represents the vertical transmission attributable to pregnant/breastfeeding women on ARVs. For 2008, this includes those who received only short‐course zidovudine or single‐dose nevirapine during pregnancy. Abbreviation: ARV, antiretroviral drug (includes three‐drug combination ART, single‐dose nevirapine or short‐course zidovudine).

CEPAC‐projected vertical transmission risks were similar to published values (Tables S2 and S3). Total projected vertical transmission (early and postpartum transmissions) declined over time in all settings. South Africa consistently had the lowest total vertical transmission. Zimbabwe experienced the greatest reduction in total vertical transmission from 2008 to 2016 (Figure 1).

In 2016, untreated pregnant/breastfeeding women with HIV were projected to comprise 24% of all pregnant/breastfeeding women with HIV in South Africa, 67% in Côte d'Ivoire and 35% in Zimbabwe, but contributed to 80% of the estimated total vertical transmission in South Africa, 95% in Côte d'Ivoire and 86% in Zimbabwe (Figure 1).

### 3.2 Projected undiagnosed prevalence

Among surviving 2‐year‐old CLWH, the projected proportion in whom HIV remained undiagnosed was 44.2% in South Africa, 55.8% in Côte d'Ivoire and 52.9% in Zimbabwe. Among the general population of 2‐year‐olds, the projected undiagnosed HIV prevalence was 0.44% in South Africa, 0.32% in Côte d'Ivoire and 0.68% in Zimbabwe. In 5‐/10‐year‐olds, the projected undiagnosed prevalence was 0.25%/0.24% in South Africa, 0.17/0.14% in Côte d'Ivoire and 0.41%/0.38% in Zimbabwe. In all countries, the projected undiagnosed prevalence was lower among older cohorts, and there was a steeper drop‐off in undiagnosed prevalence in children ageing from 2 to 5 years compared with children ageing from 5 to 10 years (Table 2). In South Africa, the absolute number of children also declined more steeply between ages 2 and 5 versus ages 5 and 10 (7% and 1% decline, respectively, Table S5).

**Table 2:** Prevalence of undiagnosed HIV among 2‐, 5‐ and 10‐year‐old children in South Africa, Côte d'Ivoire and Zimbabwe in 2018

Two‐year‐old children		
	Proportion of surviving CLWH in whom HIV not yet diagnosed, %	Undiagnosed HIV prevalence among general population, %
South Africa	44.2	0.44
Côte d'Ivoire	55.8	0.32
Zimbabwe	52.9	0.68

Five‐year‐old children		
	Proportion of surviving CLWH in whom HIV not yet diagnosed, %	Undiagnosed HIV prevalence among general population, %
South Africa	29.0	0.25
Côte d'Ivoire	37.8	0.17
Zimbabwe	33.2	0.41

Ten‐year‐old children		
	Proportion of surviving CLWH in whom HIV not yet diagnosed, %	Undiagnosed HIV prevalence among general population, %
South Africa	18.3	0.24
Côte d'Ivoire	25.4	0.14
Zimbabwe	23.1	0.38

Abbreviations: **CLWH**, children living with HIV; **HIV**, human immunodeficiency virus.

### 3.3 Projected survival and diagnosis of CLWH: 2016–2026

**Figure 2:**
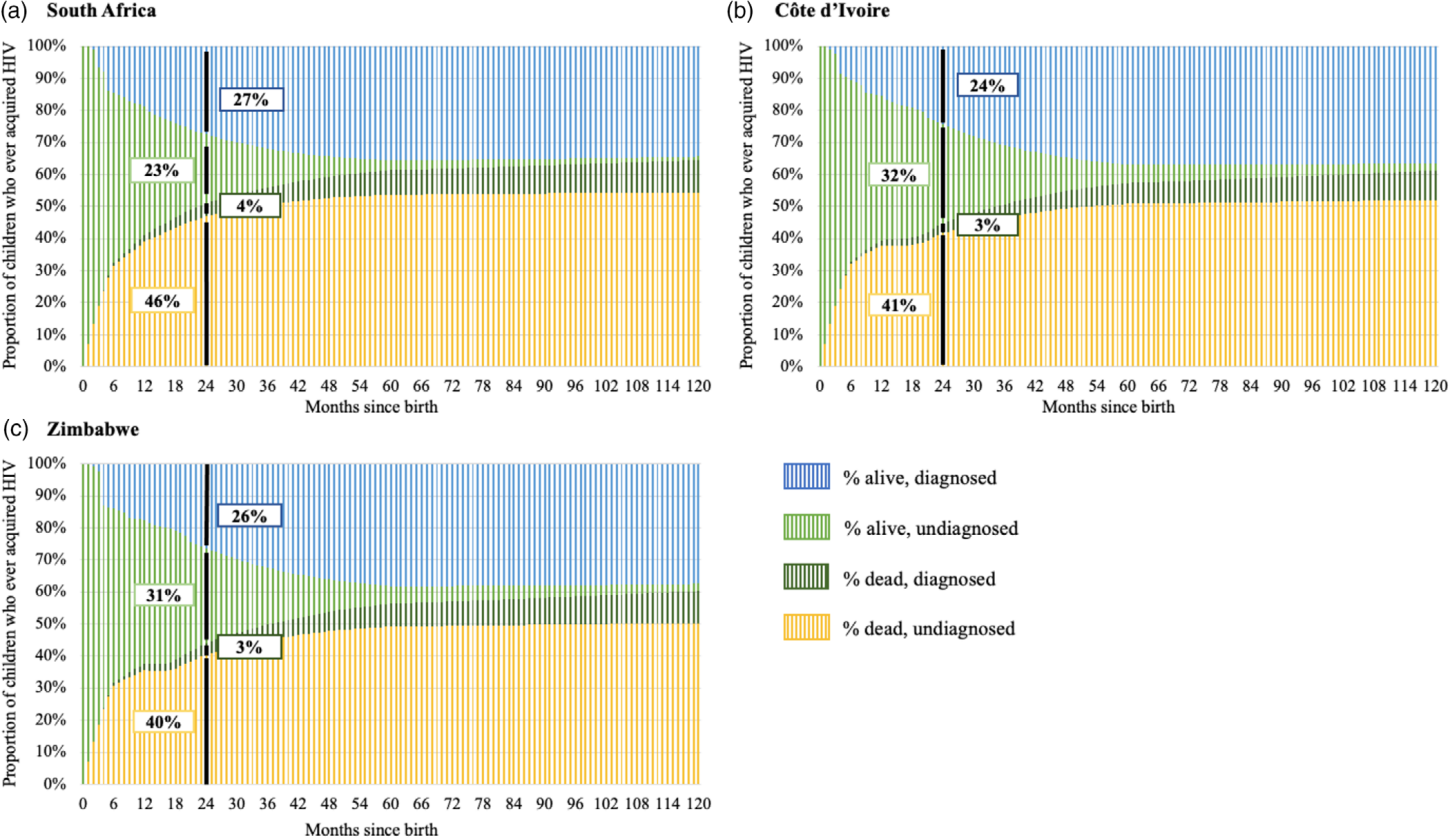
Projected 10‐year survival and diagnosis among children living with HIV born in 2016 in South Africa (Panel a), Côte d'Ivoire (Panel b) and Zimbabwe (Panel c). Projected survival and diagnosis among children living with HIV born in 2016 in (a) South Africa, (b) Côte d'Ivoire and (c) Zimbabwe, with proportion of children who ever had HIV (currently alive or dead) on the vertical axis and time since birth, in months, on the horizontal axis. For each time point, the proportion of children with HIV who remain alive and undiagnosed is shown in light green, the proportion who remain alive and diagnosed is shown in blue, the proportion who have died (of any cause) while undiagnosed is shown in yellow and the proportion who have died (of any cause) after being diagnosed is shown in dark green. The proportion of children in each category at 24 months is displayed on the chart in boxes. The spike in the number alive and diagnosed seen in Côte d'Ivoire and Zimbabwe, but not South Africa, between months 16 and 20 represents the end of breastfeeding (breastfeeding duration was shorter in South Africa). To avoid underestimating undiagnosed prevalence, model inputs from 2016 are held constant for the 2016–2026 projection. Before the first infant HIV test is conducted (usually birth and/or 6 or 10 weeks of age), all children in the CEPAC‐P model are considered to have undiagnosed HIV, even if they are receiving multi‐drug ARV prophylaxis (sometimes known as presumptive treatment). Abbreviation: **HIV**, human immunodeficiency virus.

For CLWH born in 2016, the proportion projected to survive without HIV diagnosis decreased sharply in the first 2 years of life (Figure 2, light green shading). However, this reduction was more the result of mortality (dark green and yellow) than the result of successful diagnosis (blue), with 43–50% of CLWH dying by age 2 across the modelled countries. Longer breastfeeding duration and the subsequent larger proportion of postnatal infections occurring after 6‐ or 10‐week testing led to higher proportions of children who had ever acquired HIV and were projected to remain alive with undiagnosed HIV in Côte d'Ivoire and Zimbabwe compared with South Africa (i.e. 32% and 31% in Côte d'Ivoire and Zimbabwe, respectively, compared with 23% in South Africa at 2 years; Figure 2, green boxes). By 10 years after birth, the proportion of all CLWH who were projected to have died prior to HIV diagnosis was above 50% in all three countries, with 80–85% of these deaths occurring before age 2, and 98–99% occurring before age 5. The remainder of CLWH born in 2016 survived without a diagnosis, were diagnosed and survived or were diagnosed and did not survive. The proportion of undiagnosed 10‐year‐olds is projected to be substantially lower in 2026 than in 2018 due to improvements in the HIV care continuum from 2008 to 2016 (Figure 2 and Table S6).

### 3.4 Sensitivity analyses

**Figure 3:**
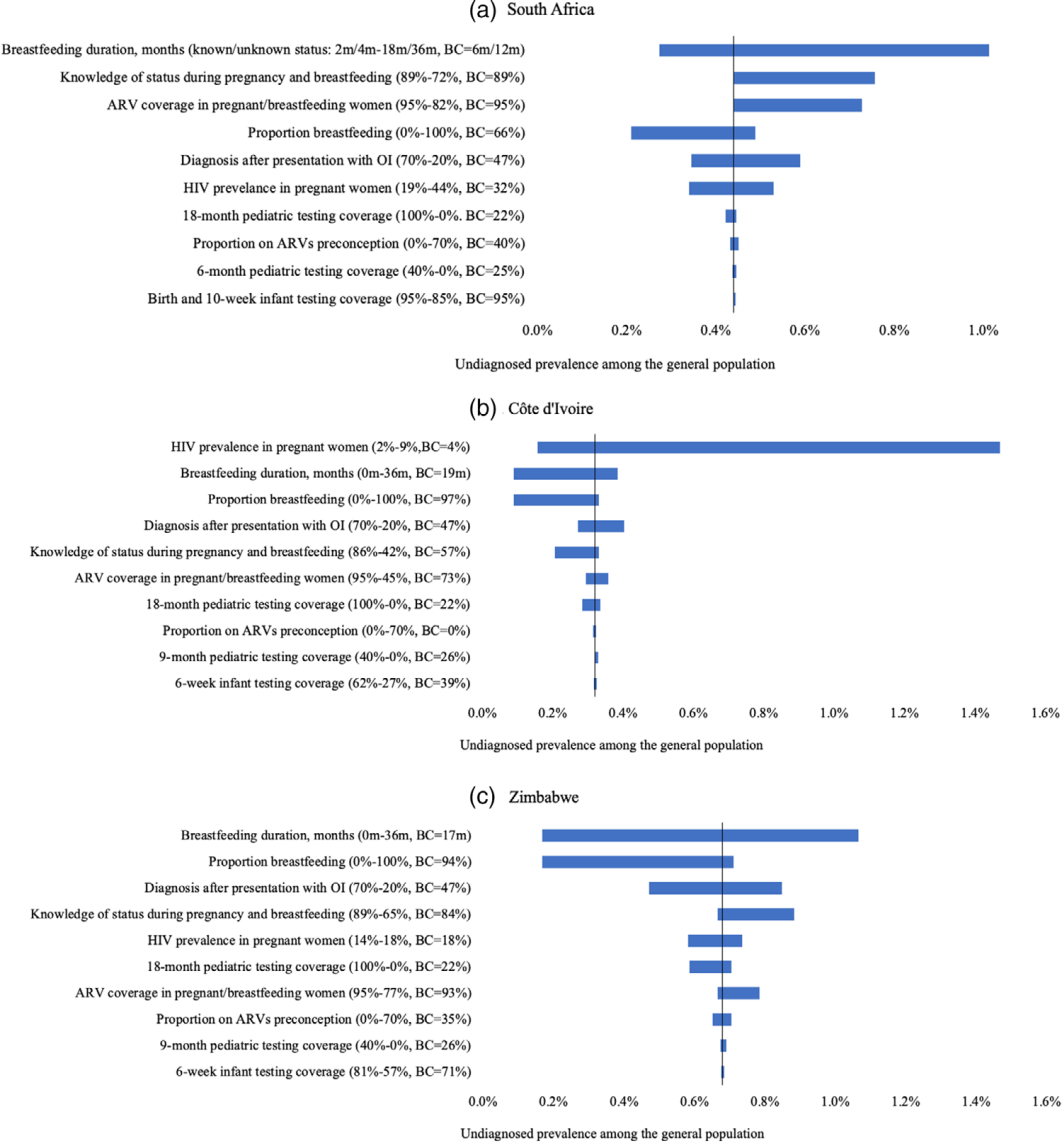
One‐way sensitivity analyses: effect of model input parameters on projected undiagnosed HIV prevalence of 2‐year‐old children in the general population in 2018 in South Africa (Panel a), Côte d'Ivoire (Panel b) and Zimbabwe (Panel c). The effect of model input parameters on undiagnosed HIV prevalence of 2‐year‐old children in 2018 is shown for (a) South Africa, (b) Côte d'Ivoire and (c) Zimbabwe, with input parameters on the vertical axis and undiagnosed HIV prevalence on the horizontal axis. For each parameter, the range of variation and base‐case values are shown in parenthesis. Abbreviations: **BC**, base‐case value; **m**, months.

When values of model input parameters were varied over plausible ranges in one‐way sensitivity analyses, breastfeeding duration had the largest impact on projected undiagnosed HIV prevalence in South Africa and Zimbabwe, and HIV prevalence in pregnant/breastfeeding women had the greatest impact in Côte d'Ivoire (Figure 3). HIV prevalence in pregnant/breastfeeding women in Côte d'Ivoire had the largest impact across all countries when varied across the plausible range (2–9%, base‐case: 4%), with a projected prevalence of undiagnosed HIV among 2‐year‐olds in the general population varying from 0.16% to 1.47% (base‐case: 0.32%). When HIV prevalence in pregnant/breastfeeding women was increased in Côte d'Ivoire, the vertical transmission was affected more than in the other settings with higher ARV coverage due to more vertical transmission from untreated women. Results from additional sensitivity analyses are in Table S4.

In multi‐way sensitivity analyses, shown in Figures S2–S4, we project scenarios in which undiagnosed HIV prevalence in 2‐year‐olds would reach specific values (range: 0.2–1.4%; depending on country‐specific variations in undiagnosed prevalence). Undiagnosed prevalence was a maximum of 1.4% in South Africa, 0.5% in Côte d'Ivoire and 1.3% in Zimbabwe, with specific combinations of knowledge of HIV status, ARV coverage and probability of diagnosis after the presentation to care with OI when these parameters were varied across country‐specific plausible ranges.

## 4 DISCUSSION

Our model‐based analysis projected that undiagnosed HIV prevalence among children aged 2, 5 and 10 years is generally low in South Africa (0.24–0.44%), Côte d'Ivoire (0.14–0.32%) and Zimbabwe (0.38–0.68%), with a steeper decline in undiagnosed prevalence between ages 2 and 5 than between ages 5 and 10. However, many CLWH surviving to age 2 remain undiagnosed: 44% in South Africa, 56% in Côte d'Ivoire and 53% in Zimbabwe. Moreover, more than 50% of all CLWH in modelled countries are projected to die by age 10 without ever having their HIV diagnosed and subsequently treated, with most deaths occurring within the first 2 years of life. Results are consistent with data from the Zimbabwe Population‐based HIV Impact Assessment, which reports that among CLWH aged 1–4, 5–9 and 10–14 years, 47%, 36% and 23% have undiagnosed HIV, respectively [38].

We find that while the HIV care continuum for women and children has improved dramatically over time, there is room for improvement in identifying CLWH, particularly in the first 2 years of life. In all settings, countries should scale up paediatric HIV testing at higher‐yield sites or deploy targeted strategies, such as index testing. We project that even when only 24% of the total population of pregnant/breastfeeding women with HIV go untreated, these women contribute 80% of total vertical transmission, consistent with prior findings [39]. This highlights the importance of improving HIV testing and linkage to ART among women in antenatal/postnatal care. Despite WHO guidelines recommending universal HIV testing of pregnant/breastfeeding women, testing coverage remains variable across countries/regions [40]. Even when testing coverage is relatively high, our sensitivity analyses show that further improving knowledge of HIV status has a substantial impact on reducing undiagnosed prevalence. Additionally, results suggest that because pregnant/breastfeeding women who are not on ART are likely to not be engaged with HIV services, testing children for HIV outside of formal infant testing programmes is critical [41, 42, 43]. Programmes offering routine, provider‐initiated HIV testing in medical settings outside of traditional pathways, such as nutrition, immunization, inpatient and TB clinics, have been found effective in identifying undiagnosed CLWH [41, 43, 44]. A recent study offering routine HIV testing for infants up to 24 months of age in Uganda found that nutrition clinics have the highest undiagnosed HIV prevalence (9.8%), followed by traditional infant testing (3.8%), inpatient (3.5%), outreach (1.7%) and immunization (0.8%) settings, highlighting that settings frequented by younger and/or sicker children will have higher undiagnosed prevalence than general population settings (e.g. households or schools) [41, 42, 43]. Still, HIV testing coverage in high‐yield sites may be low [36]; programmes should focus on maximizing coverage at these sites to increase the number of CLWH diagnosed. More research is warranted to identify settings outside of traditional infant testing to optimize HIV testing service allocation. For example, recent work has demonstrated the feasibility and cost‐effectiveness of screening to identify maternal HIV status and capture CLWH in outpatient and/or immunization settings (“screen‐and‐test”) [44, 45]. Similar to index testing, screen‐and‐test identifies the mother and infant in need of diagnostic services and can be an approach to increasing yield in outreach, outpatient or immunization settings.

In some settings, routine screening of the general population of children could be cost‐effective, especially at younger ages. A recent analysis found that PITC would be cost‐effective in South Africa if the undiagnosed prevalence is at least 0.2–0.3%; our analysis projects that undiagnosed prevalence in South Africa in 2018 is 0.24–0.44%, depending on age [15]. While a similar cost‐effectiveness analysis is not available for Zimbabwe or Côte d'Ivoire, these findings suggest that routine screening in community‐based settings could be cost‐effective for children aged 2–10 in South Africa, a high‐prevalence setting. Thus, for young children in all settings and for children of all ages in high‐prevalence settings, countries or localities could consider implementing routine screening, such as household or school‐based testing [46, 47, 48]. However, cost‐effectiveness is only one aspect of decision‐making; affordability and implementation constraints could limit routine testing feasibility. In such contexts, the implementation of routine testing could be a longer‐term goal for programmes and funders.

Our sensitivity analysis results can inform which settings should be prioritized for HIV testing of pregnant/breastfeeding women and children. For example, because lower knowledge of HIV status in pregnant/breastfeeding women is associated with higher paediatric undiagnosed HIV prevalence, subnational locations with limited access to or engagement in antenatal/postnatal care could implement PITC in settings, such as well‐child visits or community‐wide outreach programmes. HIV diagnosis during late pregnancy and breastfeeding is also critical; the optimal location, timing and frequency of repeated HIV screening among pregnant/breastfeeding women is an area of active investigation [39, 42]. Importantly, while longer breastfeeding led to higher projected undiagnosed prevalence, this analysis did not consider non‐HIV‐related breastfeeding benefits; results should not be interpreted as a recommendation to shorten breastfeeding.

Our study has several limitations. First, there are limited data against which we can validate total vertical transmission. One strength of our model‐based analysis is that it can simulate outcomes, including total vertical HIV transmission, for women and children missed by traditional testing programmes. This differentiates our analysis from clinical trials and data collection conducted primarily in antenatal care or infant testing clinics, but long‐term model‐based estimates are inherently uncertain. Notably, likely due to our estimates about low re‐testing rates in pregnancy/breastfeeding, CEPAC‐P estimates of yearly vertical transmission are generally higher than UNAIDS estimates. Second, our estimate of the proportion of women with known HIV status assumes that available data encompass all testing done during pregnancy/breastfeeding; if additional testing is done at delivery or during breastfeeding, undiagnosed prevalence will likely be lower [27, 28, 29]. Third, we do not account for suboptimal ARV regimen uptake. For example, in 2013, three‐drug ART was newly recommended for all pregnant/breastfeeding women, regardless of CD4 count. Some programmes lacked immediate access to ART for all women and may have offered short‐course zidovudine or single‐dose nevirapine instead, even after 2013. We accounted for this in sensitivity analyses by varying ARV coverage in pregnant/breastfeeding women, which revealed higher undiagnosed HIV prevalence among children in settings with lower coverage, but did not change conclusions. Our model did not specifically account for the possibility of long‐term non‐progressors among CLWH [49]. However, even when the probability of presenting to care with OI or death from chronic AIDS was reduced by 50%, undiagnosed HIV prevalence remained low. We also did not account for changes in the HIV care continuum over time within each birth cohort, due to model structure limitations and uncertainty of such changes; however, if parameters improve over time, as expected, undiagnosed prevalence would be even lower than current estimates. Lastly, we did not model specific subnational settings in which HIV care continuum parameters deviate from national averages, but a range of plausible values was examined in sensitivity analysis to represent various subnational settings.

## 5 CONCLUSIONS

In this simulation model‐based analysis, we projected that undiagnosed HIV prevalence among 2‐, 5‐ and 10‐year‐old children in the general population in South Africa, Côte d'Ivoire and Zimbabwe is low, and if ART and HIV care continuum parameters in pregnancy/breastfeeding improve over time, the undiagnosed prevalence may continue to decrease. We project that undiagnosed prevalence declines more steeply between ages 2 and 5 than between ages 5 and 10, and that more than half of CLWH die before HIV is diagnosed, with most of these deaths occurring in the first 2 years of life. We conclude that in addition to strengthening traditional infant HIV testing, programmes should: (1) prioritize deploying and maximizing coverage of innovative targeted testing in all settings, including testing of sick children/children exhibiting OI symptoms (with expected yield highest in TB, nutrition and inpatient clinics), and index testing or screen‐and‐test (e.g. in outpatient, immunization or general population settings), and (2) depending on feasibility, consider implementing routine testing of the general population of children below 2 in all settings and children of all ages in high‐prevalence settings (e.g. in households [all ages] or schools [older ages]).

## COMPETING INTERESTS

The authors have no competing interests to disclose.

## AUTHORS’ CONTRIBUTIONS

All authors have made substantial contributions to the conception and design, acquisition of data, and/or analysis and interpretation of the data. The authors have participated in drafting or critically revising this article. The authors have given final approval of the submitted version.

## FUNDING

This work was supported by the World Health Organization, with additional support for model development from the US National Institutes of Health and R01HD079214 (Ciaranello), K08HD094638 (Neilan), K08HD101342 (Dugdale) and U01AI069924 (Davies and Johnson).

## Supporting information

Supporting information
The Supplemental Technical Appendix provides additional details about the CEPAC‐P model structure and analysis‐specific supporting information.

## Data Availability

Data used to initially populate and calibrate the CEPAC‐P model were shared under data use agreements. No primary data were collected for this analysis.
