# Supplementary material for: Prevalence of undiagnosed HIV among children in South Africa, Côte d'Ivoire and Zimbabwe: a model‐based analysis to inform paediatric HIV screening programmes
Source: J Int AIDS Soc. 2022 Dec 15;25(12):e26045. doi: 10.1002/jia2.26045 (PMC9753158; doi:10.1002/jia2.26045)
Supplement: Supplementary file 1 — Supporting information The Supplemental Technical Appendix provides additional details about the CEPAC‐P model structure and analysis‐specific supporting information. [file JIA2-25-e26045-s001.docx]

**Prevalence of undiagnosed HIV among children in South Africa, Côte d’Ivoire, and Zimbabwe: a model-based analysis to inform pediatric HIV screening programs**

Supplemental Technical Appendix

Nicole C. McCann

*Et al.*

**Contents**

Introduction p.2

Methods: model structure

1. Cohort characteristics of pregnant women and vertical transmission 3
2. Calibration and validation of vertical transmission risk and pediatric diagnoses 3
3. Untreated pediatric HIV infection 3-4
4. Infant/pediatric HIV diagnosis 4
5. Treated pediatric HIV infection 4-5

Methods: Model outcomes 5

Methods: Data sources 5

Methods: Input calculations

1. Prevalence and incidence of HIV in pregnancy and breastfeeding 5
2. Probability of known HIV status in pregnant and breastfeeding women 5
3. Probability of ARV initiation preconception 5

Results 6

Table S1: Model input parameters 7-13

Table S2: South Africa IU/IP vertical transmission model calibration 14

Table S3: CEPAC-projected vertical transmission compared with UNAIDS estimates 15

Table S4: One-way sensitivity analyses: undiagnosed HIV prevalence among two-year-old children

in the general population in 2018 16-17

Table S5: Total number of two- five-, and ten-year old children with undiagnosed HIV 18

Table S6: Comparison of age-specific undiagnosed prevalence by birth cohort 19

Figure S1. CEPAC-Pediatric model state transition diagram 20

Legend: Figure S1 20

Figure S2: Multi-way sensitivity analysis: South Africa 21

Figure S3: Multi-way sensitivity analysis: Côte d’Ivoire 22

Figure S4: Multi-way sensitivity analysis: Zimbabwe 23

Legend: Figures S2-S4 24

**Introduction**

This technical appendix is included to provide methodologic details to supplement the methods description in the manuscript text, as well as additional model output and results. For reader convenience, this appendix summarizes information about the CEPAC model structure that has also been described in the technical appendices of prior publications (1–5).

**Methods**

Model structure

We have previously reported on the structure of the CEPAC-Pediatric (CEPAC-P) natural history model, reflecting HIV disease progression in the absence of antiretroviral therapy (ART) (4). The CEPAC-P model has also previously been calibrated to fit observed survival and opportunistic infection (OI) data for children and adults living with HIV, both on and off ART treatment (4–6). These calibration analyses matched CEPAC output to empiric data in the following domains: OI and survival data for children off ART, and OI and survival data for adults off and on ART (4,5,7–11). Full details of model structure, data sources, and procedures for initiating new collaborative projects are also available on the CEPAC website, at <https://www.massgeneral.org/medicine/mpec/about>. Here, we describe key model features from these prior publications and provide additional detail specific to this analysis.

*Cohort characteristics of pregnant women and vertical transmission*

The CEPAC-P model simulates a cohort of infants from birth through death. At the start of each simulation, each infant is assigned a set of characteristics from their mother, including HIV status. Among chronically or acutely infected women with HIV, we specify the probability of HIV testing and availability of antiretroviral drug regimens (ARV) during pregnancy and breastfeeding. Among women not infected during pregnancy, we model probability of acute infection during breastfeeding. HIV disease status in pregnant/breastfeeding women (chronically or acutely infected) and use of ARVs determine the risk of HIV transmission during three time periods: intrauterine ([IU]one-time risk), intrapartum ([IP]one-time risk), and postpartum ([PP]monthly risk until weaning). Acute HIV, lower ARV coverage, and later ARV initiation are associated with a higher probability of earlier vertical transmission.

ARV types for pregnant/breastfeeding women are stratified into three categories: 1) single-dose nevirapine (sdNVP), 2) short-course zidovudine (scAZT), and 3) three-drug combination antiretroviral therapy (ART). Specific ARV medication class for pregnant/breastfeeding women is not modeled; vertical transmission risks are derived from a systematic review of observational data, capturing diversity of ARV types used and adherence among pregnant/breastfeeding women at modeled time points (12). Vertical transmission risks are varied in sensitivity analysis to account for uncertainty of ARV types and adherence in pregnant/breastfeeding women (Table S4).

The CEPAC-P model takes timing of ART initiation relative to timing of infection in pregnant/breastfeeding women into account in three ways: 1) through separating vertical transmission risks by maternal acute and chronic HIV status, 2) through differential maternal ART initiation probabilities based on acute and chronic HIV status (vertical transmission risks vary based on whether a pregnant/breastfeeding woman has acute HIV [i.e., infected for less than three months] or chronic HIV [i.e., infected for more than three months], and 3) through different probabilities of knowledge of HIV status and subsequently ART initiation based on whether a mother has acute or chronic HIV; if a pregnant/breastfeeding woman is infected with HIV for longer than three months (i.e., has chronic HIV), she is more likely to be receiving ARVs. See Table S1 for year- and country- specific inputs for knowledge of HIV status stratified by acute/chronic maternal HIV.

*Calibration and validation of vertical transmission risk and pediatric diagnoses*

We calibrated our model to published IU/IP vertical transmission values from South Africa in 2008-2016 (Table S2). We calibrated to South Africa data only because published data from 2008-2016 in Côte d’Ivoire and Zimbabwe were not available. We selected base case HIV care continuum input values from a range of published data points, and ultimately chose the inputs that best predicted the published IU/IP vertical transmission output.

To accurately estimate the number of children with HIV being tested each year, we calibrated our 2008 and 2013 birth cohorts to Thembisa model output reporting the proportion of surviving children living with HIV (CLWH) with undiagnosed HIV in South Africa (13). To do this, we adjusted the probability of diagnosis after presentation to care with severe OI for the 2008 and 2013 cohorts, as there was no available published data for this parameter from these years.

Additionally, we compared our vertical transmission model output with UNAIDS estimates for all three countries. We compared our model output for total vertical transmission (including IU, IP, and PP transmission) to UNAIDS data on vertical transmission risk, which is generated by the Spectrum model (Table S3).

*Untreated pediatric HIV infection*

At the time of infant HIV infection, infants draw from distributions of HIV RNA and CD4 levels; the model uses CD4 percentage (CD4%) for children <5 years old and absolute CD4 count thereafter. Current age and CD4%/count in each month determine the risks of disease progression, including development of acute OIs and death. Without effective ART, CD4%/count declines monthly. The model tracks true CD4%/count and HIV RNA level, although clinical decisions are made based on observed information, such as symptomatic illness or observed CD4%/count or RNA levels (measured according to user-specified laboratory monitoring strategies).

*Infant/pediatric HIV diagnosis*

In the CEPAC model, infants with known HIV exposure can be diagnosed with HIV through infant and pediatric testing up to 24 months of age. In this analysis, infants can be tested at birth and ten weeks, six weeks, six or nine months, and 18 months. Probability of presenting to these infant/pediatric testing visits are based on country- and year-specific data (Table 1, Table S1).

Infants can also be diagnosed with HIV through presentation to care with an OI and subsequent HIV testing (Table 1, Table S1). The probability of diagnosis after presentation to care with an OI is modeled to be 47% in all countries in 2016, derived from observational data which found that 53% of children presenting to care with symptomatic HIV disease were not subsequently tested for HIV (14). This value was consistent with country- and program-level data reported informally to World Health Organization co-authors. To account for uncertainty, the probability of diagnosis after presentation to care with OI was varied from 20%-70% in sensitivity analysis (results shown in Table S4). This parameter is substantially more uncertain for the 2008 and 2013 cohorts due to lack of data. Therefore, in 2008 and 2013, this input was used to calibrate to projections of proportion of undiagnosed children living with HIV from the Thembisa model (13) (see *Calibration and validation of vertical transmission risk and pediatric diagnoses*, pp.3). Infants who receive a positive HIV test face a probability of initiating ART.

*Treated pediatric HIV infection*

The model includes criteria by which children can initiate first-line ART, including age, observed CD4%/count, and/or development of OIs. In the base-case of this analysis in all years, all children ≤24 months of age months can initiate ART, regardless of CD4 count, based on WHO guidelines. In 2008, children >24 months can only initiate if they have CD4 <20% (15). In 2013 and 2016, children of all ages can initiate ART, regardless of CD4 count (16). In each month, children can remain in care or be lost to follow-up; if they are lost to follow-up, they are assumed to stop ART. Children are assumed to return to care if a severe OI occurs, at which point ART is resumed.

For each ART regimen, we specify an “efficacy,” defined as the probability of suppressing HIV RNA to <400 copies/mL (c/mL), and the time point by which this occurs (24 weeks). Each regimen also confers gains in CD4%/count each month for children with suppressed HIV RNA. We also incorporate a reduction in mortality and OI risks for children on ART, independent of CD4 level and HIV RNA suppression, as observed in adults; this parameter was used for model calibration (17,18).

Children who initially suppress HIV RNA at 24 or 48 weeks face a monthly risk of virologic failure thereafter (“late failure”). Following virologic failure, HIV RNA slowly rises to a “set point” that is determined as a function of HIV RNA level at initial infection. After virologic failure, there is a user-specified (base case: 12-month) delay until CD4%/count begins to decline at pre-ART rates, leading to increased monthly risks of OIs and death, until the next effective available ART regimen is initiated.

Treatment failure in CEPAC-P can be observed through meeting clinical failure (i.e., experiencing a certain number and/or type of OIs), immunologic failure (i.e., decline in CD4%/count), or virologic failure (i.e., increase in HIV RNA) criteria. The user specifies the type and frequency of monitoring for treatment failure and confirmatory testing to confirm treatment failure. After observed treatment failure, patients can be switched to the next available line of therapy.

In this analysis, detection and confirmation of first-line ART failure was only possible after more than 24 weeks on ART and was modeled using the criteria below depending on year-specific guidelines:

- Virologic failure: Observed RNA >1,000 copies/mL, confirmed by a second RNA test at least 1 month after the first (used in all years).
- Immunologic failure: Observed CD4% <10% (for children <5 years old) or CD4 count <100/μL (for children ≥5 years old), confirmed by a second CD4%/CD4 count test at least 1 month after the first (used in 2008 and 2013).
- Clinical failure: Observed new or recurring WHO Stage 3 or 4 OI or TB event, confirmed with a CD4%/CD4 count test at least 1 month after the clinical event (used in 2008 and 2013).

When immunologic or clinical failure were observed, the ART regimen was not switched until failure was confirmed with a subsequent virologic test.

Model outcomes

For each simulated infant, the model tracks clinical events, changes in CD4%/count, and the amount of time spent in each health state. After an individual patient has died, the next infant enters the model. Large cohort sizes (30 million) are simulated in order to reduce stochastic variation and generate stable model outcomes. Once the entire cohort has been simulated, summary statistics are tallied, including number and type of clinical events and the proportion alive each month, including those with diagnosed and undiagnosed HIV. For this analysis, reported monthly outcomes include number of CLWH diagnosed and not diagnosed, and survival.

Data sources

For each country and year, we obtained data from published sources which are cited in Table 1 and Table S1. Additional inputs, including those informing sensitivity analysis ranges, are also reported in Table S1.

Input calculations

*Prevalence and incidence of HIV in pregnancy and breastfeeding*

HIV prevalence in pregnant women in each country and year was derived from published data (19–22). While reports from South Africa provide prevalence among pregnant women specifically, these data were not available for Côte d’Ivoire and Zimbabwe. Instead, we used available data on the prevalence of HIV among women aged 15-49 because data describing the relationship between HIV prevalence in pregnant and non-pregnant populations for these countries are limited (23). To address uncertainty in prevalence data, we varied HIV prevalence in pregnant women widely based on published values: from 19%-44% in South Africa (base-case=32%), from 2%-9% in Côte d’Ivoire (base-case=4%), and from years after birth, the proportion of 14%-18% in Zimbabwe (base-case=18%), shown in Figure 3 and Table S4. Incidence was derived separately during pregnancy and during breastfeeding. Incidence during pregnancy was entered into the model as a nine-month probability of having acute infection, which was then added to published values of chronic HIV prevalence to report the overall prevalence (note that chronic and acute infection during pregnancy confer different vertical transmission risks). Maternal HIV incidence during breastfeeding was entered as a monthly probability. Because incidence data from Côte d’Ivoire were not available, we used risk ratios of maternal HIV prevalence to HIV incidence during pregnancy and breastfeeding from South Africa and applied these to Côte d’Ivoire’s maternal HIV prevalence to estimate maternal HIV incidence in that setting. In all sensitivity analyses when varying prevalence, we varied incidence proportionally.

*Probability of known HIV status in pregnant and breastfeeding women*

Knowledge of HIV status in pregnant and breastfeeding women was defined as the proportion of women who attended antenatal care multiplied by the proportion of women who received an HIV test in antenatal care.

*Probability of ARV initiation preconception*

Published data report the proportions of women who start ARVs during or before pregnancy in South Africa and Zimbabwe in 2013 and 2016, and these proportions are varied in sensitivity analysis (Table S1, Table S4). In 2008 for all countries, and for all years in Côte d’Ivoire, we assumed that all women started ARVs during pregnancy due to lack of data about ARV coverage in women before pregnancy.

*ARV coverage in pregnancy/breastfeeding*

ARV coverage in pregnancy/breastfeeding is assumed to be constant throughout pregnancy and breastfeeding, representing average ARV receipt/adherence over the course of pregnancy and breastfeeding.

**Results**

*Calibration and validation*

In calibration, our projected early vertical transmission risks matched published values (Table S2). Our results for the proportion of surviving CLWH with undiagnosed HIV closely matched projections from the Thembisa model. Thembisa projects that among CLWH age two, five, and ten in South Africa, 50%, 30%, and 20% have undiagnosed HIV, respectively (13); CEPAC projects 44%, 29%, and 18%, respectively.

CEPAC-projected values for vertical transmission were similar to UNAIDS-projected values (Table S3). Generally, CEPAC-projected values tended to be higher. In particular, in Côte d’Ivoire, our estimates fell slightly above the upper bound of the 95% confidence interval of UNAIDS estimates, likely due to differences in assumptions surrounding re-testing in pregnancy and breastfeeding.

**Table S1: Model input parameters**

| **South Africa** | | | | |
| --- | --- | --- | --- | --- |
| **HIV care continuum parameter** | **2008 value** | **2013 value** | **2016 value [range examined^1^]** | **Source** |
| HIV prevalence in pregnant women, % | 32.3 | 32.3 | 32.3 [19-44] | (19–22,24–27) |
| Maternal HIV incidence during breastfeeding, monthly, % | 0.24 | 0.24 | 0.24 | (20–22) |
| Knowledge of HIV status during pregnancy and breastfeeding, chronic HIV, % | 74 | 89 | 89 [72-89] | (26,28–31) |
| Knowledge of HIV status during pregnancy, acute HIV, % | 0 | 0 | 62 | (32) |
| ARV regimen for pregnant/breastfeeding women (CD4≤200/CD4>200) | ART/scAZT | ART/ART | ART/ART | (33–35) |
| ARV coverage in pregnant/breastfeeding women, % | 73 | 90 | 95 [82-95] | (26,36–39) |
| Of women receiving ARVs, initiated prior to pregnancy, % | 0 | 40 | 40 [0-70] | (40,41) |
| Early infant testing coverage at 6 weeks (birth + 10-weeks in 2016), % | 36 | 76 | 95 [85-95] | (25,26,42–44) |
| Late pediatric testing coverage at 6 months / 18 months, % | 0 | 0 | 25 / 22 [0-40] | (45–47) |
| Probability of diagnosis after presentation with severe OI, % | 20^2^ | 20^2^ | 47 [20-70] | (14) |
| Proportion breastfeeding, % (mean duration, mean months) |  |  |  |  |
| Unknown HIV status | 82 (17) | 66 (12) | 66 (12) [0-100 (4-36)] | (29,30,48) |
| Known HIV status | 0 (0) | 66 (6) | 66 (6) [0-100 (2-18)] | (30,48) |
| **Côte d’Ivoire** | | | | |
| **HIV care continuum parameter** | **2008 value** | **2013 value** | **2016 value [range examined^1^]** | **Source** |
| HIV prevalence in pregnant women, % | 5.9 | 4.7 | 4.2 [2-9] | (26,49) |
| Incidence during breastfeeding, monthly, % | 0.04 | 0.03 | 0.03 | (20–22,49) |
| Knowledge of status during pregnancy and breastfeeding, chronic HIV, % | 27 | 32 | 57 [42-86] | (26,31,50–53) |
| Knowledge of status during pregnancy, acute HIV, % | 0 | 0 | 0 | assumption |
| ARV regimen for pregnant/breastfeeding women (CD4≤200/CD4>200) | ART/sdNVP | ART/ART | ART/ART | (54,55) |
| ARV coverage in pregnant/breastfeeding women, % | 41 | 75 | 73 [45-95] | (26,36–38) |
| Of women receiving ARVs, initiated prior to pregnancy, % | 0 | 0 | 0 | assumption |
| Early infant testing coverage at 6 weeks, % | 6 | 12 | 39 [27-62] | (26,42) |
| Late pediatric testing coverage at 9 months / 18 months, % | 0 | 0 | 26 / 22 [0-40] | (45–47) |
| Probability of diagnosis after presentation with severe OI, % | 20^2^ | 20^2^ | 47 [20-70] | (14) |
| Proportion breastfeeding, % (mean duration, months) |  |  |  |  |
| Unknown HIV status | 98 (20) | 97 (19) | 97 (19) [0-100 (0-36)] | (51,56) |
| Known HIV status | 98 (20) | 97 (19) | 97 (19) [0-100 (0-36)] |  |

**Table S1: Model input parameters, continued**

| **Zimbabwe** | | | | |
| --- | --- | --- | --- | --- |
| **HIV care continuum parameter** | **2008 value** | **2013 value** | **2016 value [range examined^1^]** | **Source** |
| HIV prevalence in pregnant women, % | 20.3 | 18.8 | 17.9 [14-18] | (26,49) |
| Incidence during breastfeeding, monthly, % | 0.15 | 0.14 | 0.14 | (20–22,49) |
| Knowledge of status during pregnancy and breastfeeding, chronic HIV, % | 47 | 73 | 84 [69-85] | (26,31,57–59) |
| Knowledge of status during pregnancy, acute HIV, % | 0 | 0 | 0 | assumption |
| ARV regimen for pregnant/breastfeeding women (CD4≤200/CD4>200) | ART /sdNVP | ART/ART | ART/ART | (60) |
| ARV coverage in pregnant/breastfeeding women, % | 36 | 78 | 93 [77-95] | (26,36–38) |
| Of women receiving ARVs, initiated prior to pregnancy, % | 0 | 35 | 35 [0-70] | (61) |
| Early infant testing coverage at 6 weeks, % | 6 | 57 | 71[57-81] | (26,42,62) |
| Late pediatric testing coverage at 9 months / 18 months, % | 0 | 0 | 26 / 22 [0-40] | (1–5) |
| Probability of diagnosis after presentation with severe OI, % | 20^2^ | 20^2^ | 47 [20-70] | (14) |
| Proportion breastfeeding, % (mean duration, months) |  |  |  |  |
| Unknown HIV status | 98 (17) | 94 (17) | 94 (17) [0-100 (0-36)] |  |
| Known HIV status | 98 (17) | 94 (17) | 94 (17) [0-100 (0-36)] |  |

**Table S1: Model input parameters**, **continued**

| **Cohort characteristics** | **Value** | **Source** |
| --- | --- | --- |
| Infant sex (female/male, %) | 51.2/48.8 | (9) |
| Pediatric CD4% (SD) at infection | 45 (10) | (4) |
| Women with CD4 ≤ 200 cells/µL during pregnancy before ARVs, 2008, % | 25 | (63) |
| Delay between primary infant/pediatric HIV test and result receipt, months | 2 | assumption |
| Probability of infant/pediatric HIV test result return, % | 80 | assumption |
| Probability of linking to care/ART among those who tested positive, % | 71 | (64) |

**Table S1: Model input parameters**, **continued**

| **Clinical data: untreated, children living with HIV** | **Value [range examined^1^]** | **Source** |
| --- | --- | --- |
| CD4 decline^3^, per month |  |  |
| <3 months of age (CD4%, IU/IP infections only) | 4.00 | (65) |
| 3-59 months of age (CD4%, IU/IP infections only) | 0.05 | (65) |
| 0-59 months of age (CD4%, PP infections only^3^) | 0.05 | (65) |
| ≥60 months of age (CD4 cells/μL, any infection type,  range by HIV RNA) | 3-6 cells | (65) |
| Monthly risk of clinical events (range by CD4%), % |  |  |
| <60 months of age |  |  |
| WHO Stage 3 event (except tuberculosis) | 3.3-11.6 [0.1-0.5x] | (9) |
| WHO Stage 4 event (except tuberculosis) | 1.4-6.4 [0.1-0.5x] | (9) |
| Tuberculosis (any body site) | 0.5-3.8 [0.1-0.5x] | (9) |
| ≥60 months of age |  |  |
| Mild fungal infection | 1.8-3.1 [0.1-0.5x] | (65) |
| Visceral bacterial infection | 0.0-0.7 [0.1-0.5x] | (65) |
| WHO Stage 3 or 4 visceral disease | 0.0-1.4 [0.1-0.5x] | (65) |
| WHO Stage 3 or 4 mucocutaneous disease | 0.0-2.3 [0.1-0.5x] | (65) |
| Other WHO Stage 3 or 4 disease | 0.0-0.7 [0.1-0.5x] | (65) |
| Other severe disease | 0.2-1.7 [0.1-0.5x] | (65) |
| Other mild disease | 2.4 [0.1-0.5x] | (65) |
| Tuberculosis (any body site) | 0.0-1.7 [0.1-0.5x] | (65) |
| Risk of death within 30 days of clinical event, % (one-time) |  |  |
| <60 months of age |  |  |
| After WHO Stage 3 or 4 event | 13.5 [0.1-0.5x] | (7) |
| After TB event | 11.1 [0.1-0.5x] | (7) |
| ≥60 months of age |  |  |
| Mild fungal infection | 0.5 [0.1-0.5x] | (7) |
| Visceral bacterial infection | 2.9 [0.1-0.5x] | (7) |
| WHO Stage 3 or 4 visceral disease | 9.2 [0.1-0.5x] | (7) |
| WHO Stage 3 or 4 mucocutaneous disease | 2.4 [0.1-0.5x] | (7) |
| Other WHO Stage 3 or 4 disease | 20.0 [0.1-0.5x] | (7) |
| Other severe disease | 6.7 [0.1-0.5x] | (7) |
| Other mild disease | 0.4 [0.1-0.5x] | (7) |
| TB (any body site) | 1.8 [0.1-0.5x] | (7) |

**Table S1: Model input parameters**, **continued**

| **Clinical data: untreated, children living with HIV and HIV-exposed children** | **Value [range examined^1^]** | **Source** |
| --- | --- | --- |
| Monthly risk of HIV-related death (range by age, CD4, and history of prior OI), % | 0.1-40.8 [0.1-0.5x] | (9) |
| Monthly risk of infant mortality among breastfed, HIV-exposed, uninfected infants, % |  |  |
| 0-2 months | 1.0 | (8,66) |
| 3-5 months | 0.4 | (8,66) |
| 6-11 months | 0.3 | (8,66) |
| 12-17 months | 0.1 | (8,66) |
| 18-23 months | 0.1 | (8,66) |
| Monthly risk of non-AIDS related mortality (range by age in yearly intervals, sex), % |  |  |
| <12 months of age | 0.38-0.46 | (67) |
| 12-60 months of age | 0.02-0.05 | (67) |
| 5-13 years of age | 0.01-0.02 | (67) |
| 13-18 years of age | 0.01-0.02 | (67) |
| ≥18 years of age | 0.01-1.60 | (67) |

| **Clinical data: on ART, children living with HIV** | **Value** | | **Source** |
| --- | --- | --- | --- |
|  | LPV/r/ABC/3TC  (1^st^-line ART) | EFV/AZT/3TC  (2^nd^-line ART) |  |
| ART efficacy: HIV RNA <50c/mL at 24 weeks on ART, % |  |  |  |
| Ages 0-59 months | 91 | 75 | (10,11,68) |
| Ages 60+ months | 82 | 82 | (10,11,68) |
| CD4% gain/CD4 gain on suppressive ART (1^st^ 6 months, after 6 months) |  |  |  |
| Ages 0-59 months | 1.9, 0.4 | 2.2, 0.7 | (10,11,68) |
| Ages 60+ months | 77.3, 4.0 | 83.2, 4.2 | (10,11,68) |
| Probability of virologic failure after initial suppression, % |  |  |  |
| Ages 0-59 months | 0.91 | 0.91 | (10,11,68) |
| Ages 60+ months | 0.72 | 0.72 | (10,11,68) |
| **ART outcomes** | **Value** | |  |
| Relative risk reduction for patients on ART |  | |  |
| Risk of opportunistic infection (age 0-13) | 85 | | (69) |
| Risk of opportunistic infection (age 13+) | 32 | | (18) |
| Mortality risk (age 0-13) | 90 | | (69) |
| Mortality risk (age 13+, range by CD4) | 55-96 | | (18) |
| Monthly loss to follow-up after ART initiation, % | 0.2 | | (70) |
| **Vertical transmission risk, %** | **Value [range examined]** | |  |
| IU/IP, off ARVs (one-time risk) |  | |  |
| Chronic HIV | 19.7 [7.4-26.0] | | (12,71) |
| Acute HIV | 18.0 [10.7-30.5] | |  |
| IU/IP, sdNVP, (one-time risk) | 6.1 [5.0-11.6] | |  |
| IU/IP, on scAZT, (one-time risk) | 3.8 [1.8-6.8] | |  |
| IU/IP, on ART, (one-time risk) |  | |  |
| ART start before pregnancy | 0.26 [0.20-0.79] | |  |
| ART start during pregnancy | 1.4 [0.0-2.4] | |  |
| PP, off ART (monthly risk) |  | |  |
| Chronic HIV | 0.89 [0.73-2.0] | |  |
| Acute HIV | 4.61 [0.73-8.0] | |  |
| PP, on ART (monthly risk) |  | |  |
| ART start before pregnancy | 0.02 [0.01-0.04] | |  |
| ART start during pregnancy | 0.11 [0.03-0.12] | |  |

**Table S1: Model input parameters**, **continued**

^1^Sensitivity analyses were conducted for the 2016 birth cohort only in order to determine the relative impact of each parameter on undiagnosed HIV prevalence in two-year-olds

^2^Due to lack of data on this parameter from 2008 and 2013, we calibrated this value to match Thembisa model-projected values for proportion of CLWH with undiagnosed HIV in order to ensure an accurate number of children receiving tests for HIV.

^3^Slower CD4 decline in children 0-2 months of age infected during breastfeeding is associated with longer projected survival of children infected during breastfeeding compared with children infected during pregnancy or birth

Abbreviations: **HIV**: human immunodeficiency virus; **ARV**: antiretroviral drug; **IU:** intrauterine; **IP:** intrapartum,; **PP:** postpartum; **ART**: three-drug combination antiretroviral therapy; **scAZT:** short-course zidovudine; **sdNVP:** single-dose nevirapine; **OI:** opportunistic infection; **RNA**: ribonucleic acid; **AIDS**: acquired immunodeficiency syndrome; **WHO:** World Health Organization; **LPV/r/ABC/3TC:** lopinavir/ritonavir/abacavir/lamivudine; **EFV/AZT/3TC**: efavirenz/zidovudine/ lamivudine

**Table S2. South Africa IU/IP vertical transmission model calibration**

|  | **Calibration target, vertical transmission, %** | **Model output^1^, %** | **Vertical transmission type^2^** | **Source** |
| --- | --- | --- | --- | --- |
| **2008** | 8.3 | 7.4 | <6 week; IU/IP | (72) |
| **2013** | 2.3 | 2.5 | 4-12 week; IU/IP | (73) |
| **2016** | 1.1 | 1.1 | At birth; IU | (74) |

^1^Model output matches the published vertical transmission type. For example, for reported <six-week vertical transmission, we extract model output for vertical transmission at six weeks, and for reported birth IU infection, we extract model output for vertical transmission at birth.

^2^Vertical transmission type used for calibration varied by year depending on the best available data. Data were primarily available for infants born to women who were engaged in care and thus aware of their HIV status. We specifically simulated infants born to women with known HIV to match published outcomes at the time points from which the most robust data were available. The overall amount of total vertical transmission (IU/IP/PP) projected by our model is higher than published IU/IP values due to transmission from pregnant and breastfeeding women living with undiagnosed or untreated HIV. Model output for total vertical transmission compared with UNAIDS estimates are shown in Table S3 below.

Abbreviations: **IU**: intrauterine; **IP:** intrapartum; **PP**: postpartum

|  | **CEPAC output, total vertical transmission** | **UNAIDS estimates, total vertical transmission [95% confidence interval]** | **Source** |
| --- | --- | --- | --- |
| **2013** |  |  |  |
| South Africa | 8% | 8% [7.7%-8.8%] |  |
| Côte d’Ivoire | 24% | 20% [17%-23%] | (75) |
| Zimbabwe | 16% | 11% [9%-15%] |  |
| **2016** |  |  |  |
| South Africa | 5% | 5% [4.5%-5.1%] |  |
| Côte d’Ivoire | 21% | 16% [12%-18%] | (75) |
| Zimbabwe | 11% | 9% [7%-13%] |  |

**Table S3. CEPAC-projected total (IU/IP/PP) vertical transmission compared with UNAIDS estimates in South Africa, Côte d’Ivoire, and Zimbabwe**

Abbreviations: **IU**: intrauterine; **IP:** intrapartum; **PP**: postpartum

UNAIDS estimates not available for 2008

**Table S4. One-way sensitivity analyses: undiagnosed HIV prevalence among two-year-old children in the general population in 2018**

|  | **South Africa** | **Côte d’Ivoire** | **Zimbabwe** |
| --- | --- | --- | --- |
|  | **Undiagnosed prevalence, %** | **Undiagnosed prevalence, %** | **Undiagnosed prevalence, %** |
| **Base case** | 0.44 | 0.32 | 0.68 |
| **HIV prevalence in pregnant women (Base-case: South Africa = 32**.**3%, Côte d’Ivoire = 4**.**2%, Zimbabwe =17**.**9%)** | | | |
| Lower bound South Africa/Côte d’Ivoire/Zimbabwe**:** 19%/ 2% / 14% | 0.34 | 0.16 | 0.58 |
| Upper bound South Africa/Côte d’Ivoire/Zimbabwe: 44% / 9% / 18% | 0.53 | 1.47 | 0.74 |
| **Knowledge of HIV status in pregnancy and breastfeeding (Base-case: South Africa= 89%, Côte d’Ivoire = 57%, Zimbabwe = 84%)** | | | |
| Lower bound South Africa/Côte d’Ivoire/Zimbabwe: 72% / 42% / 65% | 0.76 | 0.33 | 0.88 |
| Upper bound South Africa/Côte d’Ivoire/Zimbabwe: 89% / 86% / 89% | 0.44 | 0.21 | 0.67 |
| **ARV coverage in pregnant/breastfeeding women (Base-case: South Africa = 95%, Côte d’Ivoire = 73%, Zimbabwe =93%)** | | | |
| Lower bound South Africa/Côte d’Ivoire/Zimbabwe: 82% / 45%/ 77% | 0.73 | 0.36 | 0.78 |
| Upper bound South Africa/Côte d’Ivoire/Zimbabwe: 95% / 95% / 95% | 0.44 | 0.30 | 0.67 |
| **Probability of ARV initiation pre-conception (Base-case: South Africa = 40%, Côte d’Ivoire = 0%, Zimbabwe =35%)** | | | |
| Lower bound South Africa/Côte d’Ivoire/Zimbabwe: 0% / 0%/ 0% | 0.45 | 0.32 | 0.70 |
| Upper bound South Africa/Côte d’Ivoire/Zimbabwe: 70% / 70% / 70% | 0.43 | 0.31 | 0.65 |
| **6-week/birth and 10-week infant testing coverage (Base-case: South Africa = 95%, Côte d’Ivoire = 39%, Zimbabwe =71%)** | | | |
| Lower bound South Africa/Côte d’Ivoire/Zimbabwe: 85% / 27% / 57% | 0.44 | 0.32 | 0.68 |
| Upper bound South Africa/Côte d’Ivoire/Zimbabwe: 95% / 62% / 81% | 0.44 | 0.32 | 0.68 |
| **6- or 9-month pediatric testing coverage (Base-case: South Africa = 25%, Côte d’Ivoire = 26%, Zimbabwe =26%)** | | | |
| Lower bound South Africa/Côte d’Ivoire/Zimbabwe: 0% / 0% / 0% | 0.45 | 0.33 | 0.69 |
| Upper bound South Africa/Côte d’Ivoire/Zimbabwe: 40% / 40% / 40% | 0.44 | 0.32 | 0.67 |
| **18-month pediatric testing coverage (Base-case: South Africa = 22%, Côte d’Ivoire = 22%, Zimbabwe =22%)** | | | |
| Lower bound South Africa/Côte d’Ivoire/Zimbabwe: 0% / 0% / 0% | 0.45 | 0.33 | 0.71 |
| Upper bound South Africa/Côte d’Ivoire/Zimbabwe: 100% /100% / 100% | 0.42 | 0.28 | 0.59 |
| **Breastfeeding proportion (Base-case: South Africa = 66%, Côte d’Ivoire = 97%, Zimbabwe = 94%)** | | | |
| Lower bound South Africa/Côte d’Ivoire/Zimbabwe: 0% | 0.21 | 0.09 | 0.17 |
| Upper bound South Africa/Côte d’Ivoire/Zimbabwe: 100% | 0.49 | 0.33 | 0.71 |
| **Breastfeeding duration (Base-case: South Africa = 6-12m, Côte d’Ivoire = 19m, Zimbabwe = 17m )** | | | |
| Lower bound South Africa: 2-4 months; Côte d’Ivoire/Zimbabwe: 0m | 0.27 | 0.09 | 0.17 |
| Upper bound South Africa: 18-36 months; Côte d’Ivoire/Zimbabwe: 36m | 1.01 | 0.38 | 1.07 |
| **Adding birth testing** | NA- already in base case | 0.32 | 0.68 |

**Table S4. One-way sensitivity analyses: undiagnosed HIV prevalence among two-year-old children in the general population in 2018, continued**

|  | **South Africa** | **Côte d’Ivoire** | **Zimbabwe** |
| --- | --- | --- | --- |
|  | **Undiagnosed prevalence, %** | **Undiagnosed prevalence, %** | **Undiagnosed prevalence, %** |
| **Base case** | 0.44 | 0.32 | 0.68 |
| **Probability of OI, range by age and CD4 (Base-case: 0**.**0%-11**.**6% per month)** | | | |
| 0.1x base case | 0.90 | 0.55 | 1.17 |
| 0.5x base case | 0.64 | 0.43 | 0.90 |
| **Probability of diagnosis after OI (Base-case=47%)** |  |  |  |
| Lower bound: 20% | 0.59 | 0.40 | 0.85 |
| Upper bound: 70% | 0.35 | 0.27 | 0.47 |
| **Chronic AIDS death: HIV-related death not due to OI, range by age, CD4, and history of prior OI (Base-case: 0**.**1%-40**.**8% per month)** | | | |
| 0.1x base case | 0.69 | 0.44 | 0.93 |
| 0.5x base case | 0.56 | 0.38 | 0.81 |
| **Vertical transmission risks (Base-case: IU/IP, one-time risk = 0**.**26%-19**.**7%, PP, monthly risk = 0**.**02%-0**.**89%)** | | | |
| Lower bound^1^: IU/IP=0%-10.7%, PP=0.01%-0.73% | 0.23 | 0.21 | 0.39 |
| Upper bound^1^: IU/IP= 0.79%-30.5%, PP=0.04%-8.0% | 0.67 | 0.53 | 1.10 |

Abbreviations: **ARV**: antiretroviral drug; **ART**: three-drug antiretroviral therapy; **OI:** opportunistic infection; **IU:** intrauterine; **IP:** intrapartum, **PP:** postpartum; **m:** months

**Table S5. Total number of two-, five-, and ten-year old children with undiagnosed HIV in 2018 in South Africa**

|  | **Total number of CLWH** | **Proportion of surviving CLWH in whom HIV not yet diagnosed, %** | **Total number of undiagnosed CLWH, N** |
| --- | --- | --- | --- |
| Two-year-old children | 13,000 | 44.2 | 5,746 |
| Five-year-old children | 18,500 | 29.0 | 5,365 |
| Ten-year-old children | 29,000 | 18.3 | 5,307 |

^1^ The estimates of total number of CLWH are derived from Johnson *et al., Pediatr Infect Dis J*, 2020 (13) . To calculate the total number of undiagnosed CLWH, we multiplied the model-projected proportion of surviving CLWH in whom HIV is not yet diagnosed by the total number of CLWH. We only project these absolute numbers for South Africa due to lack of single year stratified, age-specific data on total number of CLWH in Côte d’Ivoire and Zimbabwe. Projections of absolute numbers are inherently uncertain due to limited population size data.

|  | **2008 birth cohort**  (Two-year-olds, year=2010)  (Five-year-olds=, year=2013)  (Ten-year-olds, year=2018; base-case) | **2013 birth cohort**  (Two-year-olds, year=2015)  (Five-year-olds, year=2018; base-case)  (Ten-year-olds, year=2023) | **2016 birth cohort**  (Two-year-olds, year=2018; base-case)  (Five-year-olds, year=2021)  (Ten-year-olds, year=2026) |
| --- | --- | --- | --- |
| South Africa, undiagnosed prevalence in general population / in surviving CLWH | | | |
| Two-year olds | 1.83% / 73.6% | 0.92% / 70.6% | 0.44% / 44.2% |
| Five-year olds | 0.51% / 32.7% | 0.25% / 29.0% | 0.07% / 8.15% |
| Ten-year olds | 0.24% / 18.3% | 0.11% / 15.0% | 0.02% / 3.18% |
| Côte d’Ivoire, undiagnosed prevalence in general population / in surviving CLWH | | | |
| Two-year-old children | 0.86% / 80.8% | 0.55% / 79.4% | 0.32% / 55.8% |
| Five-year-old children | 0.27% / 40.9% | 0.17% / 37.8% | 0.06% / 13.3% |
| Ten-year-old children | 0.14% / 25.4% | 0.09% / 22.1% | 0.03% / 6.1% |
| Zimbabwe, undiagnosed prevalence in general population / in surviving CLWH | | | |
| Two-year-old children | 2.53% / 79.2% | 1.30% / 71.8% | 0.68% / 52.9% |
| Five-year-old children | 0.78% / 38.5% | 0.41% / 33.2% | 0.13% / 12.5% |
| Ten-year-old children | 0.38% / 23.1% | 0.20% / 19.1% | 0.05% / 5.8% |

**Table S6. Comparison of age-specific undiagnosed prevalence by birth cohort**

^1^Projections are shown for two-, five-, and ten-year olds in each birth cohort (2008, 2013, and 2016). The year of the projected undiagnosed prevalence for each age is shown in the column titles. For example, undiagnosed prevalence in two-year-old children from the 2008 birth cohort represents undiagnosed prevalence in two-year-old children in 2010. Comparisons of undiagnosed prevalence among children of the same age over time can be made across rows. For example, undiagnosed prevalence in two-year-old children (among general population / among surviving CLWH) in South Africa decreased from 1.83% / 73.6% to 0.92% / 70.6% to 0.44% / 44.2% from 2010 to 2015 to 2018, respectively. Base-case values for 2018 presented in the main manuscript are shown in red.


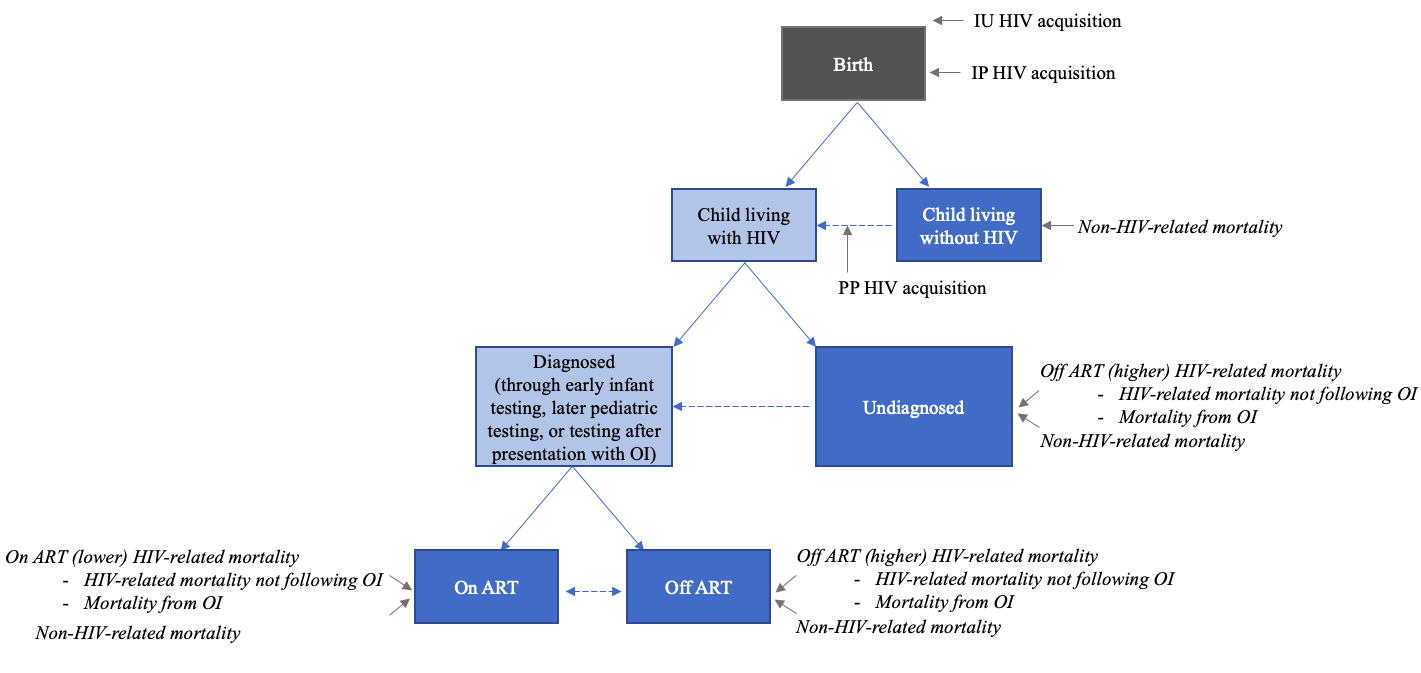
**Figure S1. CEPAC-Pediatric model state transition diagram**

**Legend: Figure S1**

Figure S1 shows state transitions for the modeled pediatric cohort in this analysis. Each dark blue box represents a possible state that simulated children can be in after birth. Blue arrows represent state transitions based on model input probabilities (dotted arrows represent possible transitions). Children face non-HIV-related mortality in all states (italicized). Children with undiagnosed or untreated HIV additionally face non-OI or OI-related HIV-related mortality; children with diagnosed and treated HIV also face HIV-related mortality but with lower risks due to ART efficacy (italicized). Additional flow charts detailing the CEPAC-Pediatric model can be found at: <https://www.massgeneral.org/assets/mgh/pdf/medicine/mpec/cepac-peds%20flowchart-08_28_2015.pdf>

**Abbreviations:** **IU:** intrauterine; **IP:** intrapartum; **PP:** postpartum; **OI:** opportunistic infection; **ART:** antiretroviral therapy; **HIV:** human immunodeficiency virus; **AIDS**: acquired immune deficiency syndrome

**
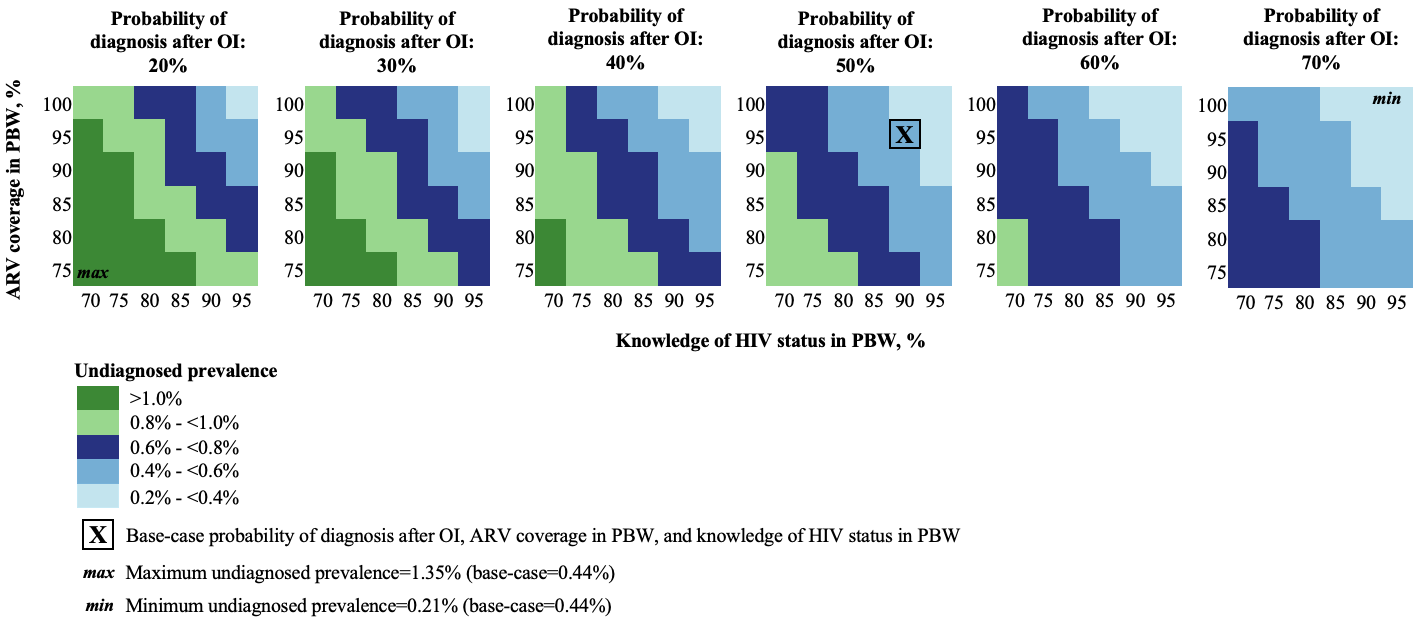
Figure S2. Multi-way sensitivity analysis: undiagnosed prevalence of HIV among two-year-old children in South Africa in the general population, 2018**

**Figure S3. Multi-way sensitivity analysis: undiagnosed prevalence of HIV among two-year-old children in Côte d’Ivoire in the general population, 2018**

**
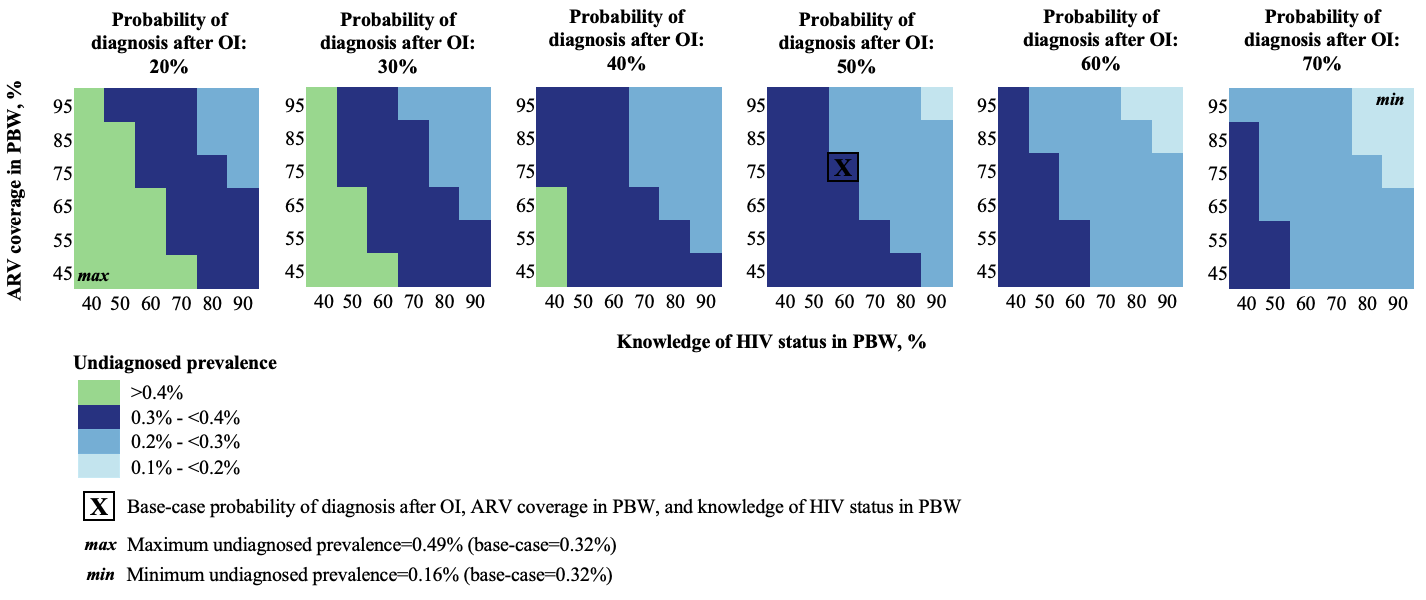
**

**Figure S4. Multi-way sensitivity analysis: undiagnosed prevalence of HIV among two-year-old children in Zimbabwe in the general
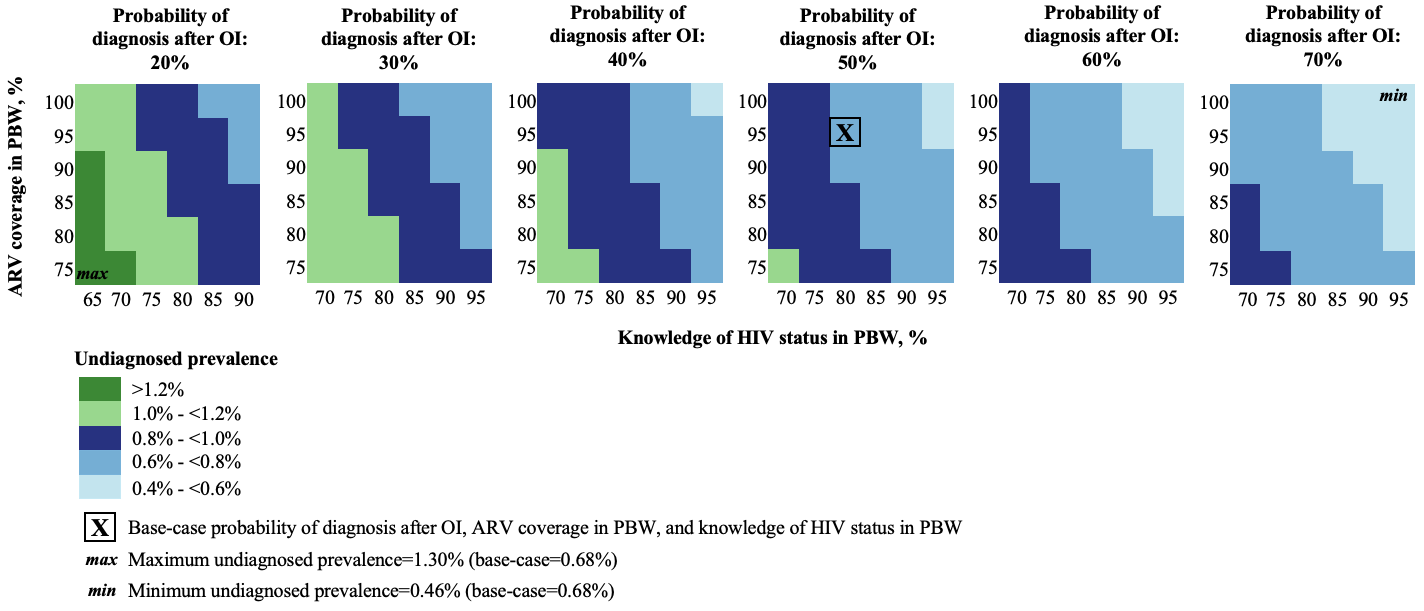
population, 2018**

**Legend: Figures S2-S4:**

Figure S2, S3, and S4 show the undiagnosed prevalence in the general population of two-year-old children when varying knowledge of HIV status in pregnant and breastfeeding women, ARV coverage in pregnant and breastfeeding women, and pediatric HIV diagnosis after presentation to care with an OI in South Africa, Côte d’Ivoire, and Zimbabwe, respectively. For each country, each parameter is varied across a plausible range. Each color represents the level of undiagnosed prevalence, as specified in the key below each figure. The base-case is marked with an X, and the maximum and minimum undiagnosed prevalence values are marked on the figure.

**Abbreviations:** **ARV**: antiretroviral drug; **OI**: opportunistic infection; **PBW**: pregnant and breastfeeding women

**REFERENCES**

1. Francke JA, Penazzato M, Hou T, Abrams EJ, MacLean RL, Myer L, et al. Clinical impact and cost-effectiveness of diagnosing HIV infection during early infancy in South Africa: test timing and frequency. J Infect Dis. 2016 Nov 1;214(9):1319–28.

2. Dunning L, Francke JA, Mallampati D, MacLean RL, Penazzato M, Hou T, et al. The value of confirmatory testing in early infant HIV diagnosis programmes in South Africa: A cost-effectiveness analysis. PLoS Med. 2017 Nov;14(11):e1002446.

3. Frank SC, Cohn J, Dunning L, Sacks E, Walensky RP, Mukherjee S, et al. Clinical effect and cost-effectiveness of incorporation of point-of-care assays into early infant HIV diagnosis programmes in Zimbabwe: a modelling study. Lancet HIV. 2019 Mar;6(3):e182–90.

4. Ciaranello AL, Morris BL, Walensky RP, Weinstein MC, Ayaya S, Doherty K, et al. Validation and calibration of a computer simulation model of pediatric HIV infection. PLoS ONE. 2013;8(12):e83389.

5. Ciaranello AL, Lockman S, Freedberg KA, Hughes M, Chu J, Currier J, et al. First-line antiretroviral therapy after single-dose nevirapine exposure in South Africa: a cost-effectiveness analysis of the OCTANE trial. AIDS. 2011 Feb 20;25(4):479–92.

6. Walensky RP, Ross EL, Kumarasamy N, Wood R, Noubary F, Paltiel AD, et al. Cost-effectiveness of HIV treatment as prevention in serodiscordant couples. N Engl J Med. 2013 Oct 31;369(18):1715–25.

7. Dabis F, Bequet L, Ekouevi DK, Viho I, Rouet F, Horo A, et al. Field efficacy of zidovudine, lamivudine and single-dose nevirapine to prevent peripartum HIV transmission. AIDS. 2005 Feb 18;19(3):309–18.

8. Marston M, Becquet R, Zaba B, Moulton LH, Gray G, Coovadia H, et al. Net survival of perinatally and postnatally HIV-infected children: a pooled analysis of individual data from sub-Saharan Africa. International Journal of Epidemiology. 2011 Apr 1;40(2):385–96.

9. Ciaranello A, Lu Z, Ayaya S, Losina E, Musick B, Vreeman R, et al. Incidence of World Health Organization stage 3 and 4 events, tuberculosis and mortality in untreated, HIV-infected children enrolling in care before 1 year of age: an IeDEA (International Epidemiologic Databases to Evaluate AIDS) East Africa regional analysis. The Pediatric Infectious Disease Journal. 2014 Jun;33(6):623–9.

10. Violari A, Cotton MF, Gibb DM, Babiker AG, Steyn J, Madhi SA, et al. Early antiretroviral therapy and mortality among HIV-infected infants. N Engl J Med. 2008 Nov 20;359(21):2233–44.

11. Palumbo P, Lindsey JC, Hughes MD, Cotton MF, Bobat R, Meyers T, et al. Antiretroviral treatment for children with peripartum nevirapine exposure. N Engl J Med. 2010 Oct 14;363(16):1510–20.

12. Stover J, Glaubius R, Mofenson L, Dugdale CM, Davies MA, Patten G, et al. Updates to the Spectrum/AIM model for estimating key HIV indicators at national and subnational levels: AIDS. 2019 Dec;33:S227–34.

13. Johnson LF, Patrick M, Stephen C, Patten G, Dorrington RE, Maskew M, et al. Steep declines in pediatric AIDS mortality in South Africa, despite poor progress toward pediatric diagnosis and treatment targets. Pediatr Infect Dis J. 2020 Sep;39(9):843–8.

14. Feucht UD, Meyer A, Thomas WN, Forsyth BWC, Kruger M. Early diagnosis is critical to ensure good outcomes in HIV-infected children: outlining barriers to care. AIDS Care. 2016;28(1):32–42.

15. World Health Organization. Report of the WHO Technical Reference Group, Paediatric HIV/ART Care Guideline Group Meeting [Internet]. 2008 [cited 2022 May 31]. Available from: https://www.who.int/hiv/pub/paediatric/WHO_Paediatric_ART_guideline_rev_mreport_2008.pdf

16. World Health Organization. Consolidated guidelines on the use of antiretroviral drugs for treating and preventing HIV infection [Internet]. 2013 [cited 2020 Jun 2]. Available from: https://www.who.int/hiv/pub/guidelines/arv2013/download/en/

17. Ciaranello AL, Lockman S, Freedberg KA, Hughes M, Chu J, Currier J, et al. First-line antiretroviral therapy after single-dose nevirapine exposure in South Africa: a cost-effectiveness analysis of the OCTANE trial: AIDS. 2011 Feb;25(4):479–92.

18. Losina E, Yazdanpanah Y, Deuffic-Burban S, Wang B, Wolf LL, Messou E, et al. The independent effect of highly active antiretroviral therapy on severe opportunistic disease incidence and mortality in HIV-infected adults in Côte d’Ivoire. Antivir Ther (Lond). 2007;12(4):543–51.

19. Dinh TH, Delaney KP, Goga A, Jackson D, Lombard C, Woldesenbet S, et al. Impact of maternal HIV seroconversion during pregnancy on early mother to child transmission of HIV (MTCT) measured at 4-8 weeks postpartum in South Africa 2011-2012: a national population-based evaluation. Davies MA, editor. PLoS ONE. 2015 May 5;10(5):e0125525.

20. Chetty T, Vandormael A, Thorne C, Coutsoudis A. Incident HIV during pregnancy and early postpartum period: a population-based cohort study in a rural area in KwaZulu-Natal, South Africa. BMC Pregnancy Childbirth. 2017 Dec;17(1):248.

21. Fatti G, Shaikh N, Jackson D, Goga A, Nachega JB, Eley B, et al. Low HIV incidence in pregnant and postpartum women receiving a community-based combination HIV prevention intervention in a high HIV incidence setting in South Africa. Paraskevis D, editor. PLoS ONE. 2017 Jul 27;12(7):e0181691.

22. le Roux SM, Abrams EJ, Nguyen KK, Myer L. HIV incidence during breastfeeding and mother-to-child transmission in Cape Town, South Africa: AIDS. 2019 Jul;33(8):1399–401.

23. Eaton JW, Rehle TM, Jooste S, Nkambule R, Kim AA, Mahy M, et al. Recent HIV prevalence trends among pregnant women and all women in sub-Saharan Africa: implications for HIV estimates. AIDS. 2014 Nov;28 Suppl 4:S507-514.

24. Mnyani CN, Buchmann EJ, Chersich MF, Frank KA, McIntyre JA. Trends in maternal deaths in HIV-infected women, on a background of changing HIV management guidelines in South Africa: 1997 to 2015. J Intern AIDS Soc. 2017 Nov;20(3):e25022.

25. South Africa National AIDS Council. South Africa Global AIDS Response Progress Report (GARPR) 2015 [Internet]. 2015 [cited 2022 May 31]. Available from: https://sanac.org.za/?page_id=2591&cfrom=01-01-2015#552-wpfd-2015

26. UNAIDS. Joint United Nations Programme on HIV/AIDS [Internet]. 2018 [cited 2022 May 31]. Available from: https://www.aidsdatahub.org/sites/default/files/publication/UNAIDS_Data_2018.pdf

27. South Africa National Department of Health. National antenatal sentinel HIV & syphilis survey report. 2015.

28. Day C, Barron P, Monticelli F, Sello E. The District Health Barometer Year 2007/08 [Internet]. Durban Health Systems Trust. [cited 2022 May 31]. Available from: https://www.hst.org.za/publications/District%20Health%20Barometers/DHB0708.pdf

29. Department of Health, Medical Research Council. South Africa Demographic and Health Survey 2003 [Internet]. 2007 [cited 2022 May 31]. Available from: https://dhsprogram.com/pubs/pdf/FR206/FR206.pdf

30. Department of Health, Republic of South Africa, STATS SA, saMRC, NDP. South Africa Demographic and Health Survey, 2016 [Internet]. 2019 [cited 2022 May 31]. Available from: https://dhsprogram.com/pubs/pdf/FR337/FR337.pdf

31. World Health Organization. HIV country intelligence: HIV country profiles [Internet]. 2017 [cited 2022 May 31]. Available from: http://cfs.hivci.org/index.html

32. de Beer S, Kalk E, Kroon M, Boulle A, Osler M, Euvrard J, et al. A longitudinal analysis of the completeness of maternal HIV testing, including repeat testing in Cape Town, South Africa. J Int AIDS Soc. 2020 Jan;23(1):e25441.

33. National Department of Health. Policy and Guidelines for the Implementation of the PMTCT Programme [Internet]. 2008 [cited 2020 Jun 2]. Available from: https://www.ilo.org/wcmsp5/groups/public/---ed_protect/---protrav/---ilo_aids/documents/legaldocument/wcms_125633.pdf

34. Department of Health, Republic of South Africa. The South African Antiretroviral Treatment Guidelines [Internet]. 2013 [cited 2020 Jun 2]. Available from: https://sahivsoc.org/Files/2013%20ART%20Treatment%20Guidelines%20Final%2025%20March%202013%20corrected.pdf

35. Department of Health, Republic of South Africa. National Consolidated Guidelines for the Prevention of Mother-to-Child-Transmission and the Management of HIV in Children, Adolescents and Adults [Internet]. 2015 [cited 2020 Jun 2]. Available from: https://sahivsoc.org/Files/ART%20Guidelines%2015052015.pdf

36. UNAIDS. Children and AIDS: Fourth Stocktaking Report [Internet]. 2009 [cited 2022 May 31]. Available from: https://www.aidsdatahub.org/sites/default/files/documents/Children_and_AIDS_Fourth_Stocktaking_Report_2009.pdf

37. UNAIDS. The Gap Report [Internet]. 2014 [cited 2022 May 31]. Available from: https://www.unaids.org/en/resources/documents/2014/20140716_UNAIDS_gap_report

38. UNAIDS. UNAIDS Data 2017 [Internet]. 2017 [cited 2022 May 31]. Available from: https://www.unaids.org/en/resources/documents/2017/2017_data_book

39. Kalk E, Kroon M, Boulle A, Osler M, Euvrard J, Stinson K, et al. Neonatal and infant diagnostic HIV-PCR uptake and associations during three sequential policy periods in Cape Town, South Africa: a longitudinal analysis. J Intern AIDS Soc. 2018 Nov;21(11):e25212.

40. Cragg CD. Evaluating viral load monitoring in antiretroviral-experienced HIV-positive pregnant women accessing antenatal care in Khayelitsha, Cape Town [Internet]. University of Cape Town; 2015 [cited 2020 Jun 2]. Available from: https://open.uct.ac.za/bitstream/handle/11427/16490/thesis_hsf_2015_cragg_carol_diane.pdf?sequence=1&isAllowed=y

41. Iyun V, Brittain K, Phillips TK, le Roux S, McIntyre JA, Zerbe A, et al. Prevalence and determinants of unplanned pregnancy in HIV-positive and HIV-negative pregnant women in Cape Town, South Africa: a cross-sectional study. BMJ Open. 2018 Apr;8(4):e019979.

42. UNAIDS. Elimination of mother-to-child transmission: early infant diagnosis [Internet]. AIDSinfo. [cited 2022 May 31]. Available from: https://aidsinfo.unaids.org/

43. Barron P, Pillay Y, Doherty T, Sherman G, Jackson D, Bhardwaj S, et al. Eliminating mother-to-child HIV transmission in South Africa. Bull World Health Organ. 2013 Jan 1;91(1):70–4.

44. UNAIDS. Country Factsheets: South Africa [Internet]. [cited 2020 Jun 2]. Available from: https://www.unaids.org/en/regionscountries/countries/southafrica

45. Onoya D, Jinga N, Nattey C, Mongwenyana C, Mngadi S, Sherman GG. Motivational interviewing retention counseling and child HIV testing in South Africa. In 2020. Available from: https://www.croiconference.org/abstract/motivational-interviewing-retention-counseling-and-child-hiv-testing-in-south-africa/

46. Massyn N, Pillay Y, Padarath A. District Health Barometer 2017/2018 [Internet]. [cited 2022 May 31]. Available from: https://www.hst.org.za/publications/Pages/DHB20172018.aspx

47. ZIMPHIA. Zimbabwe Population-Based HIV Impact Assessment 2015-2016 [Internet]. [cited 2022 May 31]. Available from: https://phia.icap.columbia.edu/wp-content/uploads/2016/11/ZIMBABWE-Factsheet.FIN_.pdf

48. Myer L, Phillips TK, Zerbe A, Brittain K, Lesosky M, Hsiao NY, et al. Integration of postpartum healthcare services for HIV-infected women and their infants in South Africa: A randomised controlled trial. Geng EH, editor. PLoS Med. 2018 Mar 30;15(3):e1002547.

49. UNAIDS. HIV prevalence: population: female adults 15-49 [Internet]. AIDSInfo. [cited 2022 May 31]. Available from: https://aidsinfo.unaids.org/

50. UNICEF. Côte d’Ivoire: PMTCT [Internet]. 2010 [cited 2022 May 31]. Available from: https://www.unicef.org/aids/files/CoteDIvoire_PMTCTFactsheet_2010.pdf

51. UNICEF. Côte d’Ivoire Multiple Indicator Monitoring Survey (MIMS) 2016 [Internet]. 2016 [cited 2022 May 31]. Available from: http://mics.unicef.org/surveys

52. Côte d’Ivoire: Enquête Démographique et de Santé et à Indicateurs Multiples 2011-2012, Ministère de la Santé et de la Lutte contre le Sida, Institut National de la Statistique, Ministère d’État, Ministère du Plan et du Développement, MEASURE DHS, ICF International. Côte d’Ivoire: Enquête Démographique et de Santé et à Indicateurs Multiples 2011-2012 [Internet]. [cited 2022 May 31]. Available from: https://www.dhsprogram.com/pubs/pdf/FR272/FR272.pdf

53. African Child Data and Statistics Portal. Africa Report on Child Wellbeing - Maternal and Child Health [Internet]. [cited 2020 Jun 2]. Available from: http://data.africanchildinfo.net/prptlzd/africa-report-on-child-wellbeing-maternal-and-child-health?tsId=1001160

54. Coffie PA, Ekouevi DK, Chaix ML, Tonwe-Gold B, Clarisse AB, Becquet R, et al. Maternal 12-month response to antiretroviral therapy following prevention of mother-to-child transmission of HIV type 1, Ivory Coast, 2003- 2006. Clinical Infectious Diseases. 2008 Feb 15;46(4):611–21.

55. Rowan BH, Robinson J, Granato A, Bla CK, Kouyaté S, Djety GV, et al. Workforce patterns in the prevention of mother to child transmission of HIV in Côte d’Ivoire: a qualitative model. Hum Resour Health. 2018 Dec;16(1):4.

56. Iliff PJ, Piwoz EG, Tavengwa NV, Zunguza CD, Marinda ET, Nathoo KJ, et al. Early exclusive breastfeeding reduces the risk of postnatal HIV-1 transmission and increases HIV-free survival: AIDS. 2005 Apr;19(7):699–708.

57. UNICEF. Zimbabwe Multiple Indicator Monitoring Survey (MIMS) 2014 [Internet]. 2015 [cited 2022 May 31]. Available from: http://mics.unicef.org/surveys

58. Zimbabwe National Statistics Agency. Zimbabwe Demographic and Health Survey 2015 [Internet]. 2016 [cited 2022 May 31]. Available from: https://dhsprogram.com/pubs/pdf/FR322/FR322.pdf

59. UNICEF. Zimbabwe Multiple Indicator Monitoring Survey (MIMS) 2009 [Internet]. 2010 [cited 2022 May 31]. Available from: http://mics.unicef.org/surveys

60. Ciaranello AL, Perez F, Keatinge J, Park JE, Engelsmann B, Maruva M, et al. What will it take to eliminate pediatric HIV? Reaching WHO target rates of mother-to-child HIV transmission in Zimbabwe: a model-based analysis. Binagwaho A, editor. PLoS Med. 2012 Jan 10;9(1):e1001156.

61. Dinh TH, Mushavi A, Shiraishi RW, Tippett Barr B, Balachandra S, Shambira G, et al. Impact of timing of antiretroviral treatment and birth weight on mother-to-child human immunodeficiency virus transmission: findings from an 18-Month prospective cohort of a nationally representative sample of mother–infant pairs during the transition from Option A to Option B+ in Zimbabwe. Clinical Infectious Diseases. 2018 Feb 1;66(4):576–85.

62. Madziro NT, Mugurungi O, Chirenda J, Mungati M, Bangure D, Gombe TN, et al. Analysis of the national early infant diagnosis dataset, Zimbabwe: 2007 to 2010. J AIDS HIV Res. 2015 Jul 31;7(6):61–7.

63. Carter RJ, Dugan K, El-Sadr WM, Myer L, Otieno J, Pungpapong N, et al. CD4+ cell count testing more effective than HIV disease clinical staging in identifying rregnant and postpartum women eligible for antiretroviral therapy in resource-limited settings: JAIDS Journal of Acquired Immune Deficiency Syndromes. 2010 Nov;55(3):404–10.

64. Hsiao NY, Stinson K, Myer L. Linkage of HIV-infected infants from diagnosis to antiretroviral therapy services across the Western Cape, South Africa. John-Stewart GC, editor. PLoS ONE. 2013 Feb 6;8(2):e55308.

65. Holmes CB, Wood R, Badri M, Zilber S, Wang B, Maartens G, et al. CD4 decline and incidence of opportunistic infections in Cape Town, South Africa: implications for prophylaxis and treatment. JAIDS Journal of Acquired Immune Deficiency Syndromes. 2006 Aug;42(4):464–9.

66. Becquet R, Marston M, Dabis F, Moulton LH, Gray G, Coovadia HM, et al. Children who acquire HIV infection perinatally are at higher risk of early death than those acquiring infection through breastmilk: a meta-analysis. Bhutta ZA, editor. PLoS ONE. 2012 Feb 23;7(2):e28510.

67. United Nations. World Population Prospects: The 2008 Revision New York 2009 [Internet]. [cited 2020 Jun 2]. Available from: World Population Prospects: The 2008 Revision New York 2009

68. Walmsley SL, Antela A, Clumeck N, Duiculescu D, Eberhard A, Gutiérrez F, et al. Dolutegravir plus abacavir–lamivudine for the treatment of HIV-1 infection. N Engl J Med. 2013 Nov 7;369(19):1807–18.

69. Ciaranello AL, Doherty K, Penazzato M, Lindsey JC, Harrison L, Kelly K, et al. Cost-effectiveness of first-line antiretroviral therapy for HIV-infected African children less than 3 years of age: AIDS. 2015 Jun;29(10):1247–59.

70. Ciaranello AL, Chang Y, Margulis AV, Bernstein A, Bassett IV, Losina E, et al. Effectiveness of pediatric antiretroviral therapy in resource‐limited settings: a systematic review and meta‐analysis. CLIN INFECT DIS. 2009 Dec 15;49(12):1915–27.

71. Ciaranello AL, Perez F, Engelsmann B, Walensky RP, Mushavi A, Rusibamayila A, et al. Cost-effectiveness of World Health Organization 2010 guidelines for prevention of mother-to-child HIV transmission in Zimbabwe. Clin Infect Dis. 2013 Feb;56(3):430–46.

72. Sherman GG, Rivka LR. Early infant diagnosis of HIV infection in South Africa: 2008 to 2010 [Internet]. 2011 [cited 2020 Jun 2]. Available from: https://www.nhls.ac.za/wp-content/uploads/2020/01/EID_HIV_PCR_2008-2010.pdf

73. Sherman GG, Mazanderani AH, Barron P, Bhardwaj S, Niit R, Okobi M, et al. Toward elimination of mother–to–child transmission of HIV in South Africa: how best to monitor early infant infections within the Prevention of Mother–to–Child Transmission Program. Journal of Global Health. 2017 Jun;7(1):010701.

74. Moyo F, Haeri Mazanderani A, Barron P, Bhardwaj S, Goga AE, Pillay Y, et al. Introduction of routine HIV birth testing in the South African National Consolidated Guidelines: The Pediatric Infectious Disease Journal. 2018 Jun;37(6):559–63.

75. UNAIDS, AIDSinfo. Mother-to-child transmission rate [Internet]. [cited 2021 Mar 8]. Available from: https://aidsinfo.unaids.org/
